# Supplementary material for: eIF4A inactivates TORC1 in response to amino acid starvation
Source: EMBO J. 2016 Mar 17;35(10):1058–76. doi: 10.15252/embj.201593118 (PMC4868951; doi:10.15252/embj.201593118)
Supplement: Supplementary file 1 — Appendix [file EMBJ-35-1058-s001.pdf]

APPENDIX

eIF4A inactivates TORC1
in response to amino acid starvation

Foivos-Filippos Tsokanos[1,*], Marie-Astrid Albert[1,*], Constantinos Demetriades[1,*], Kerstin Spirohn[2], Michael Boutros[2] and Aurelio A. Teleman[1]

[1]German Cancer Research Center (DKFZ), Division of Signal Transduction in Cancer and Metabolism, 69120 Heidelberg, Germany

[2]German Cancer Research Center (DKFZ), Division of Signaling and Functional Genomics and Heidelberg University, Dept. Cell and Molecular Biology, Medical Faculty Mannheim, 69120 Heidelberg, Germany

Appendix Table of Contents

Appendix Figures S1 – S5

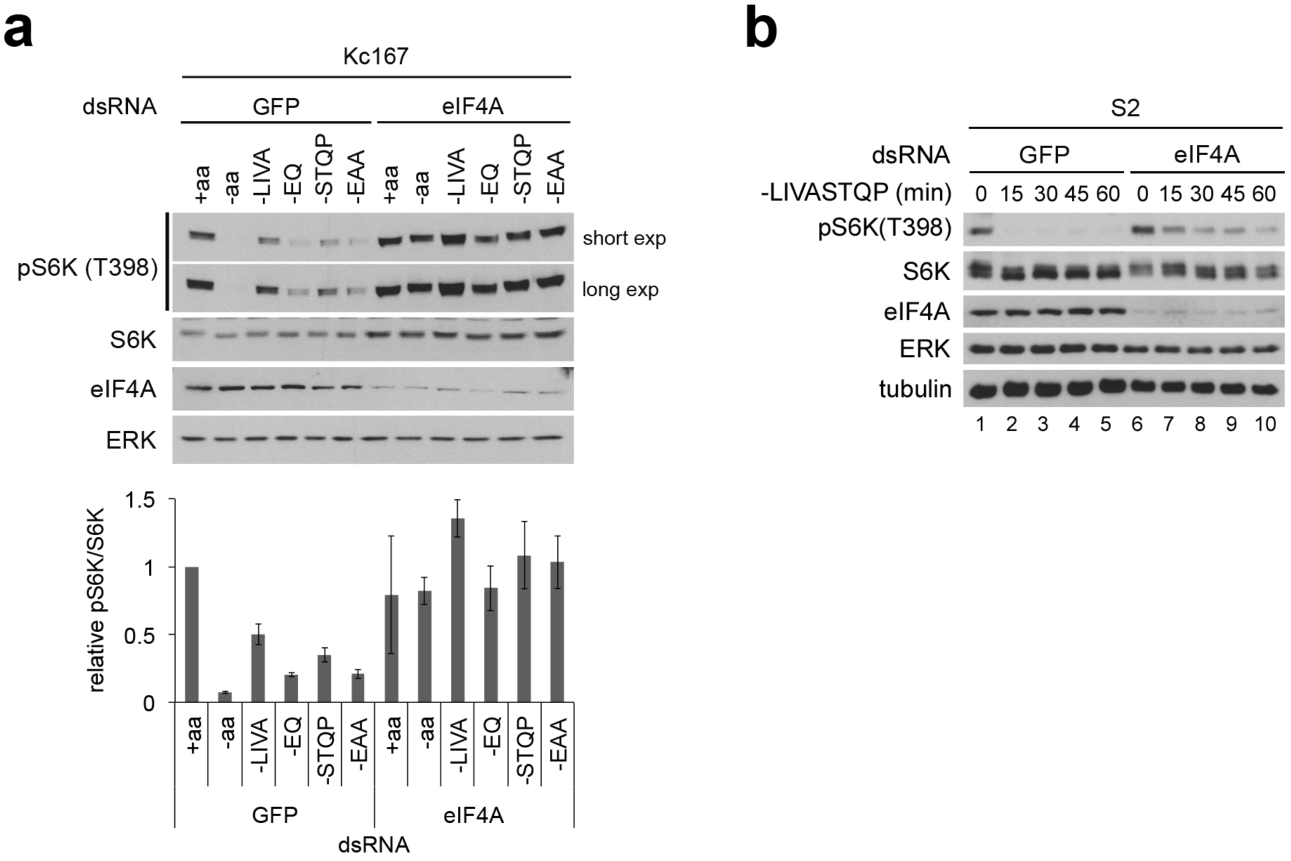

Appendix Figure S1

(a) Same as Figure 1d, but using Kc167 cells. Impaired inactivation of TORC1 in response to eIF4A knockdown is most apparent upon partial depletion of amino acids, caused by removal of amino acid subsets. After 5 days of knockdown, Kc167 cells were treated for 30 min with either complete Schneider's medium (+aa), Schneider's medium lacking all amino acids (-aa) or various subsets of amino acids, as indicated (where EAA "essential amino acids" = H,I,L,K,M,T,W,V). Error bars: Std dev. n=3 biological replicates.

(b) Same as in Figure 1e, but using S2 cells. Timecourse of amino-acid removal reveals that eIF4A knockdown S2 cells maintain elevated S6K

phosphorylation from 15 to 45 minutes of amino acid removal. Representative of two biological replicates.

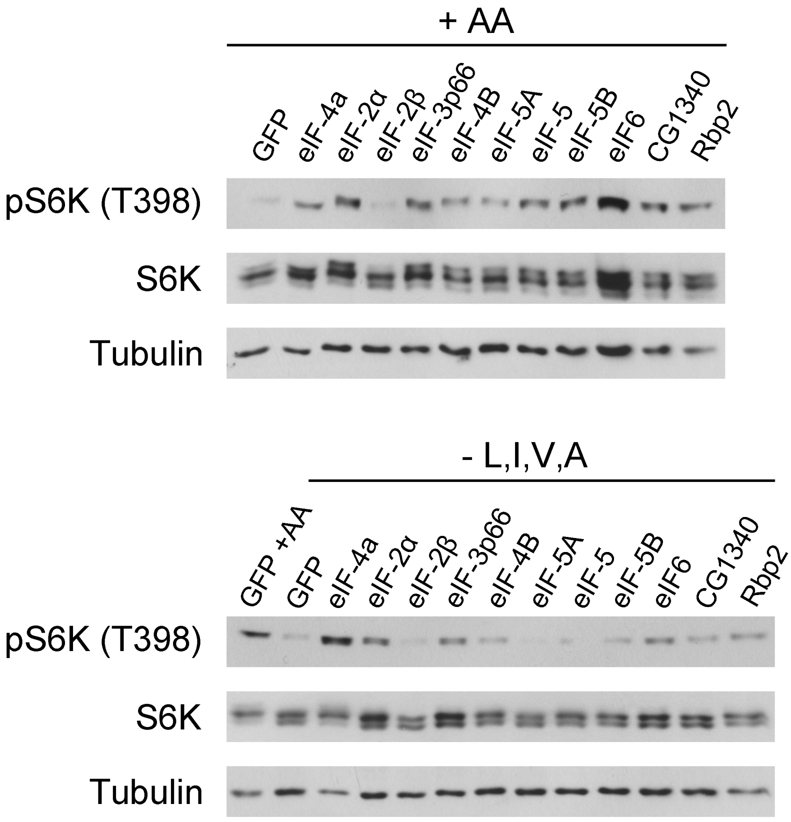

Appendix Figure S2

Same as Figure 2b, but using S2 cells. Knockdown of eIF4A, but not other translation initiation factors, blunts TORC1 inactivation upon amino acid withdrawal. S2 cells treated with indicated dsRNAs for 4 days and then incubated with complete Schneider's medium or Schneider's medium lacking the indicated amino acids for 30 minutes prior to lysis.

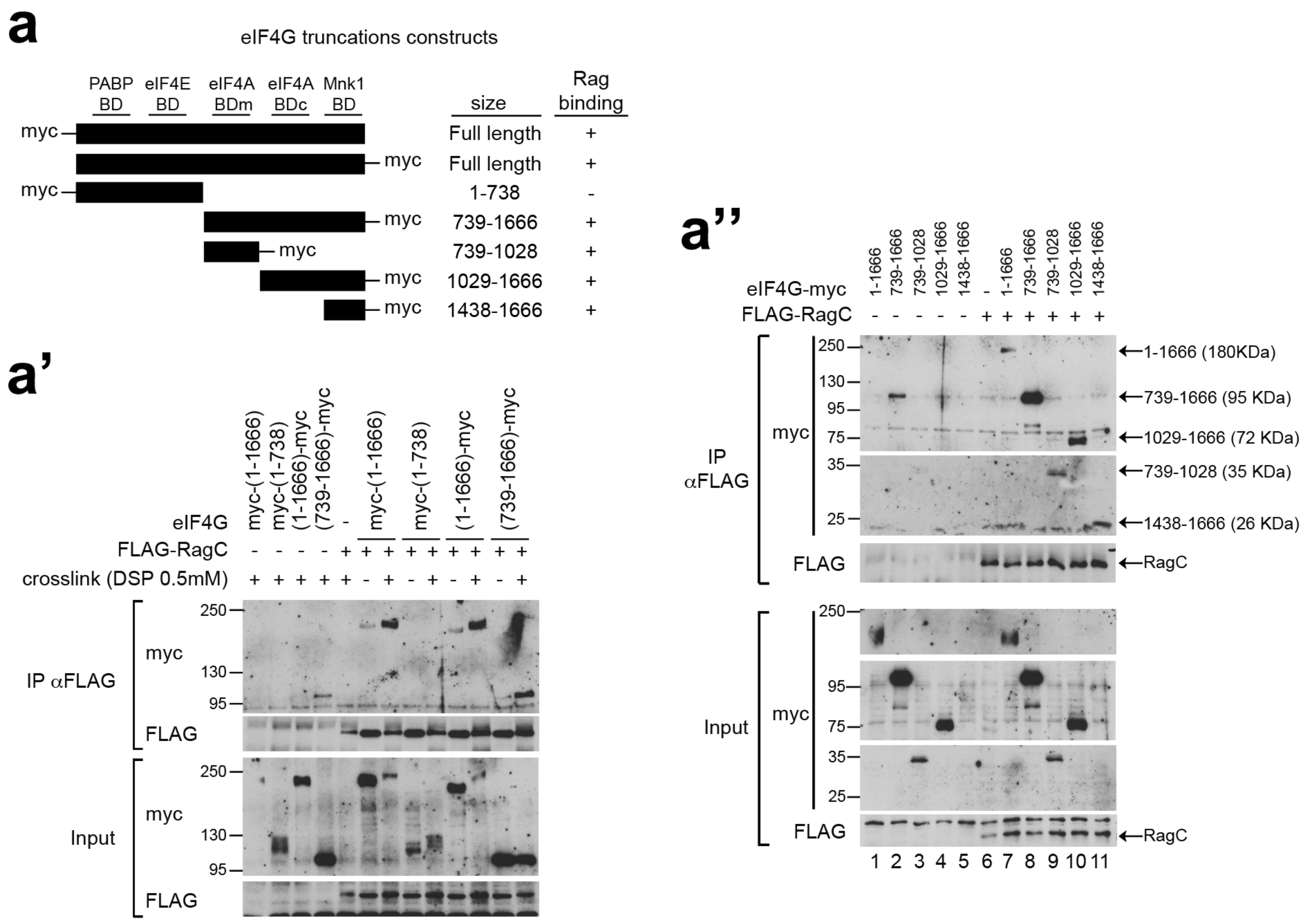

Appendix Figure S3

(a-a'') The C-terminal 229 amino acids of eIF4G bind RagC. (a) Schematic diagram of the eIF4G truncations tested. Structural domains of eIF4G are indicated at the top: poly-A binding protein binding domain (PABP-BD), eIF4E binding domain (eIF4E BD), two independent eIF4A binding domains (eIF4A BDm and BDc) and the Mnk1 binding domain (Mnk1 BD). (a') The C-terminal half of eIF4G binds RagC, tested by co-immunoprecipitation of FLAG-RagC. (a'') eIF4G amino acids 1438-1666 are sufficient to bind RagC. DSP (0.5 mM)

was added 7 minutes prior to lysis to cross-link proteins. Representative of two biological replicates.

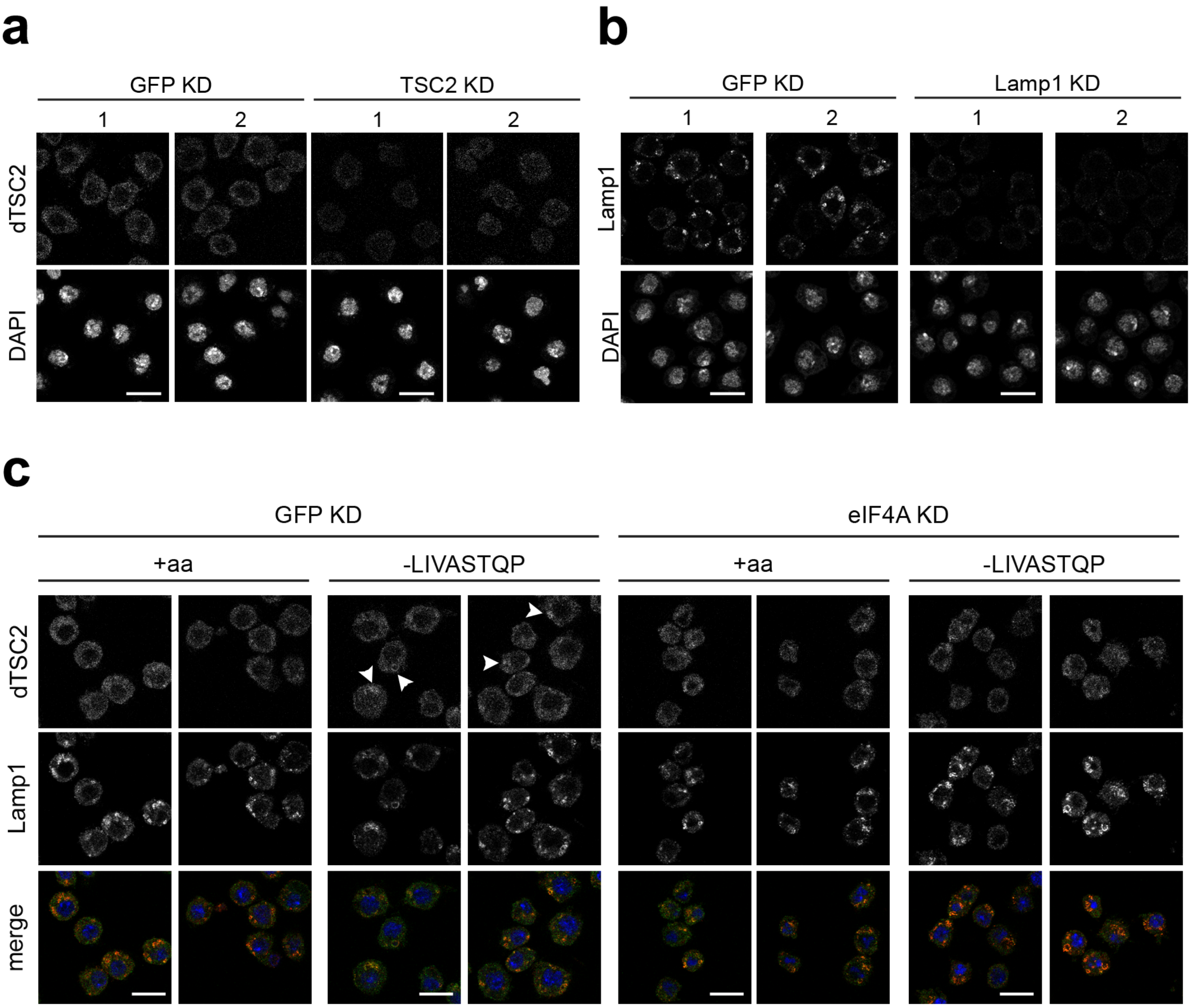

Appendix Figure S4

(a) Specificity of TSC2 antibody staining verified by knocking down endogenous TSC2 with dsRNA in Kc167 cells. Scale bars: 10µm.

(b) Specificity of Lamp1 antibody staining verified by knocking down endogenous Lamp1 with dsRNA in Kc167 cells. Scale bars: 10µm.

(c) eIF4A knockdown does not obviously alter TSC2 subcellular localization. In control cells (GFP knockdown, left panels), TSC2 is mainly cytosolic in the

presence of amino acids, with mild accumulation on lysosomes, labeled with Lamp1. Upon amino acid removal for 30 min, lysosomal accumulations of TSC2 become more prominent (arrowheads). Knockdown of eIF4A (right panels) does not appreciably affect TSC2 subcellular localization. Nuclei are labeled with DAPI (blue labeling in merge fields). Scale bars: 10μm.

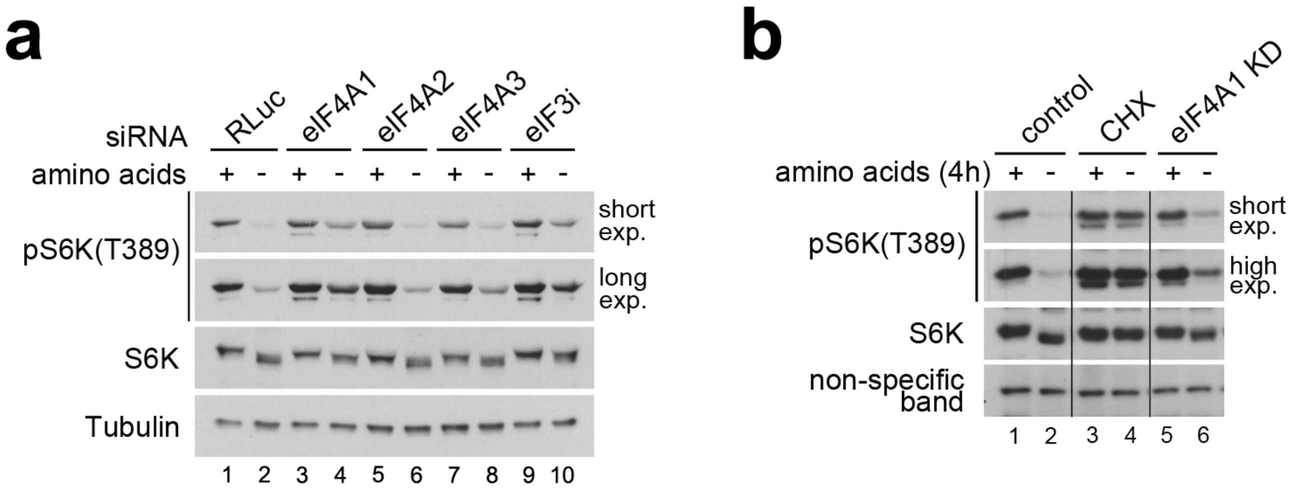

Appendix Figure S5

(a-b) Although eIF4A1 knockdown in HeLa cells causes elevated TORC1 activity upon amino acid removal compare to control knockdown (siRLuc) (a-b), other means of blocking translation such as eIF3i knockdown (a) or treatment with cycloheximide (b), also lead to elevated TORC1 activity in HeLa cells upon amino acid removal. HeLa cells treated with indicated siRNAs for 3 days were treated with DMEM containing or lacking all amino acids for 4 hours prior to lysis and immunoblotting. Where indicated, cells were treated with CHX (50µg/ml) for 5min before and during incubation with DMEM containing or lacking all amino acids. Representative of 2 biological replicates.
