# Supplementary material for: eIF4A inactivates TORC1 in response to amino acid starvation
Source: EMBO J. 2016 Mar 17;35(10):1058–76. doi: 10.15252/embj.201593118 (PMC4868951; doi:10.15252/embj.201593118)
Supplement: Supplementary file 2 — Expanded View Figures PDF [file EMBJ-35-1058-s002.pdf]

Expanded View Figures

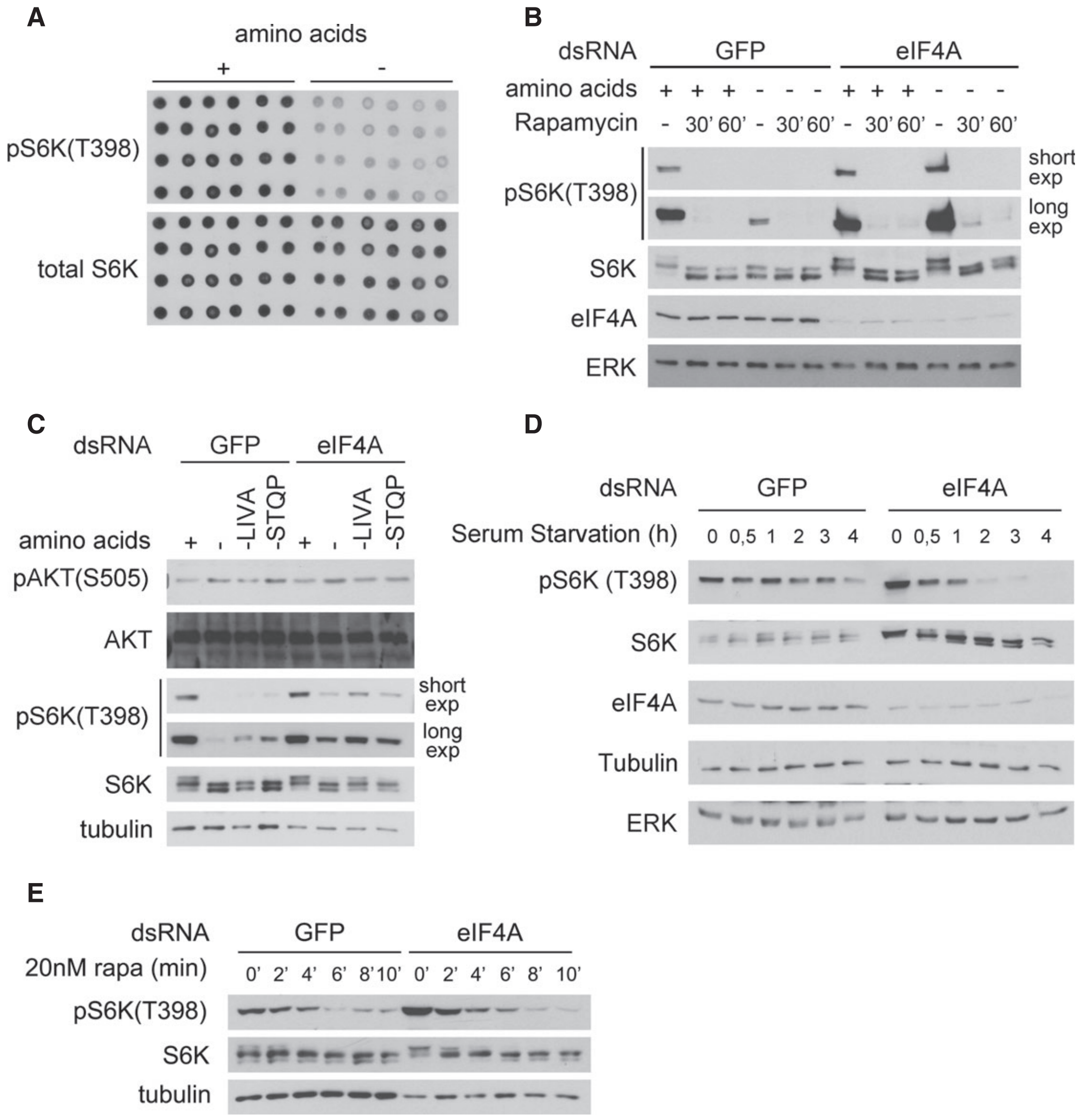

Figure EV1. eIF4a knockdown causes cells to retain elevated TORC1 activity upon amino acid removal.

- A S6K phosphorylation can be detected in cell lysates by dot-blot analysis. Lysates of S2 cells treated with Schneider's medium containing (+) or lacking (–) amino acids for 30 min, spotted onto nitrocellulose membranes, and probed with the indicated antibodies.
- B The elevated S6K phosphorylation observed upon eIF4A knockdown is caused by elevated TORC1 activity, since it can be abolished with rapamycin treatment. Kc167 cells were incubated for 5 days with negative control GFP or eIF4a dsRNA. Cells were then pretreated for 5 or 30 min with rapamycin (20 nM) followed by incubation in Schneider's medium + 10% dialyzed FBS with or without amino acids for 30 min in the presence or absence of rapamycin (20 nM). Total rapamycin treatment times (pretreatment + incubation) are therefore 30 or 60 min as indicated. Representative of two biological replicates.
- C eIF4A knockdown does not affect TORC2 activity, detected using phosphorylation of Akt on the TORC2 site (Ser505). Kc167 cells were incubated 5 days with the indicated dsRNAs, then treated for 30 min with media containing 10% dialyzed FBS and containing or lacking the indicated amino acids. Representative of three biological replicates.
- D eIF4A knockdown does not cause insensitivity to serum removal. Kc167 cells were treated for 4 days with either control GFP or eIF4A dsRNA. Cells were then treated with Schneider's medium lacking FBS for the indicated times.
- E eIF4A knockdown does not lead to reduced phosphatase activity toward S6K. A high-resolution time course of rapamycin treatment on Kc167 cells reveals that the rate of S6K dephosphorylation in control cells and eIF4A-knockdown cells is similar. (If anything, the rate of dephosphorylation in eIF4A-knockdown cells is higher than in control cells, since eIF4A-knockdown cells start with very high pS6K levels.) Representative of two biological replicates.

© 2016 The Authors

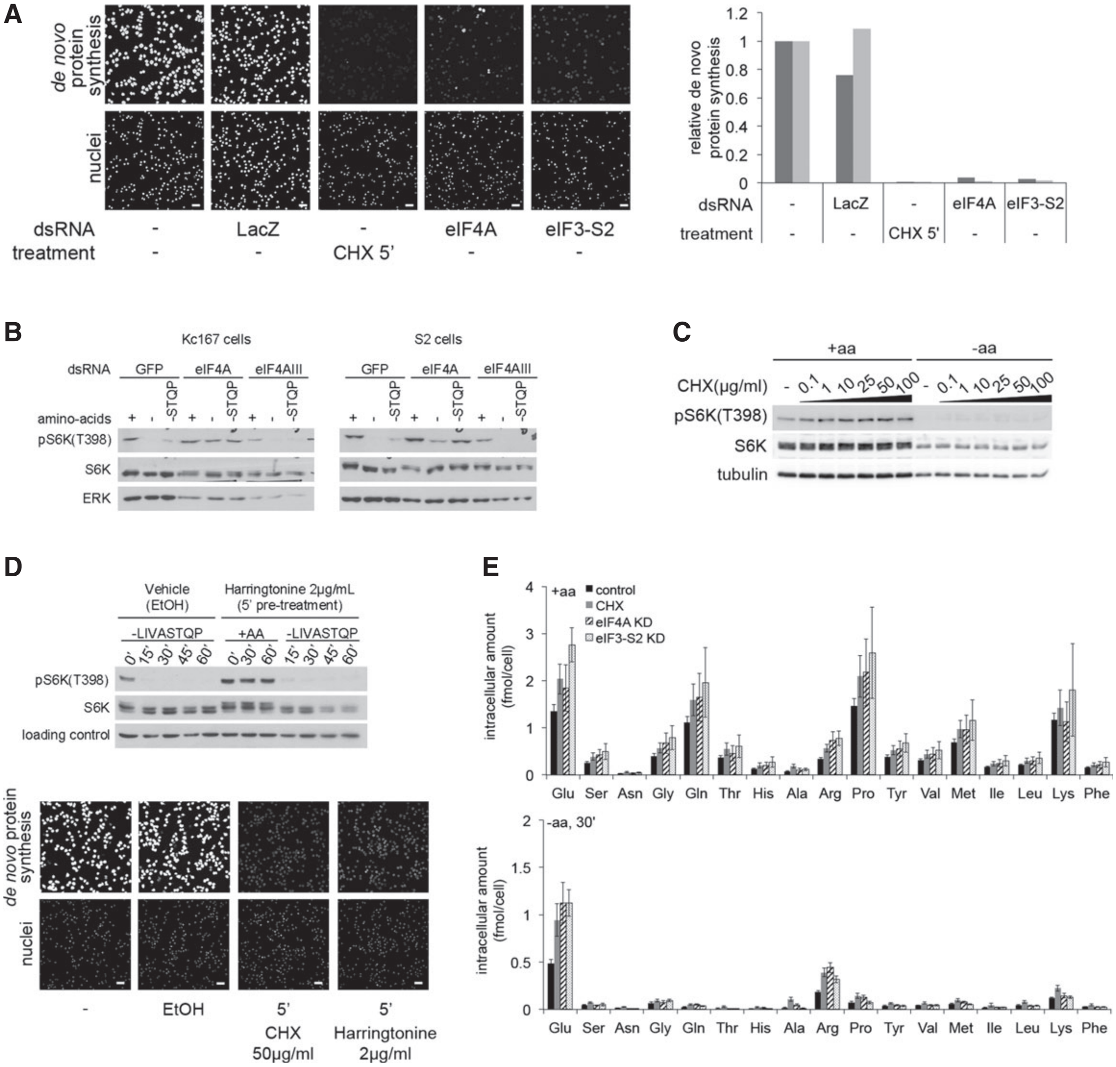

Figure EV2. Elevated TORC1 activity upon eIF4A knockdown is not a general response to translation inhibition.

A Quantification of *de novo* protein synthesis rates by OPP incorporation reveals that eIF4A knockdown does not block translation more efficiently than eIF3-S2 knockdown or cycloheximide (CHX). Kc167 cells treated with CHX (50 μg/ml) for 5 min or dsRNA against eIF4A or eIF3-S2 for 4 days were incubated with 20 μM Click-it OPP reagent for 30 min before fixation and fluorescence labeling. Quantification of OPP fluorescence per cell (nuclear count) for two independent experiments is displayed (three independent images per condition), normalized to the no dsRNA condition. Scale bars: 25 μm.

B Elevated TORC1 activity upon amino acid removal is a phenotype specific to eIF4A knockdown and is not observed upon knockdown of the highly homologous gene eIF4AIII, involved in splicing. Representative of three biological replicates.

C Blocking translation with cycloheximide does not prevent TORC1 activity from dropping in *Drosophila* S2 cells upon the removal of amino acids. Titration curve of cycloheximide is shown; 10 μg/ml cycloheximide is already sufficient to block translation and leads to elevated TORC1 activity in the +aa condition.

D Harringtonine (2 μg/ml) blocks translation, visualized via incorporation of OPP into nascent chains, but does not prevent TORC1 activity from dropping in *Drosophila* Kc167 cells upon the removal of amino acids. Cells were treated with cycloheximide (50 μg/ml) or harringtonine (2 μg/ml) for 5 min before and during treatment with media either containing (+aa) or lacking (-LIVASTQP) amino acids. OPP assay: Kc167 cells treated with CHX (50 μg/ml) or harringtonine (2 μg/ml) for 5 min were incubated with 20 μM Click-it OPP reagent for 30 min before fixation and fluorescence labeling. Scale bars: 25 μm. Representative of two biological replicates.

E Knockdown of eIF4A does not prevent a drop in intracellular amino acids when amino acids are removed from the medium for 30 min. Quantification of individual intracellular amino acids shown here. Sum of all amino acids shown in main Fig 2D. For CHX samples, cycloheximide (50 μg/ml) was added 5 min prior to, and during treatment with medium containing or lacking amino acids. Error bars indicate SD. *n* = 5 replicates.

© 2016 The Authors

Figure EV3. Analysis of possible modes of TORC1 regulation by eIF4A.

A–C″ Knockdown of eIF4A does not influence TORC1 activity via ATP levels or TORC1 complex dissociation. (A) ATP levels drop upon the removal of amino acids from the medium in Kc167 and S2 cells treated with either eIF4A dsRNA or non-targeting negative control GFP dsRNA. The ATP levels were normalized to total protein levels, quantified by Bradford reagent. Error bars indicate SD. $n = 3$. (B) Amino acid removal does not cause dissociation of TORC1 dimers. TORC1 dimerization assayed by co-immunoprecipitation of myc-Raptor with FLAG-Raptor. Kc167 cells overexpressing FLAG and/or myc-Raptor were treated for 30 min with the indicated media, followed by cross-linking with DSP and lysis in CHAPS-containing buffer. Representative of two biological replicates. (C) Amino acid removal does not cause the disruption of TORC1 complexes, assayed by detecting endogenous Raptor co-immunoprecipitating with endogenous TOR in Kc167 cells. Kc167 cells were starved for amino acids for 45 min (lane 2), or incubated with 10 μg/ml insulin for 60 min (lane 3), or 20 nM rapamycin for 30 min (lane 4), before lysis in CHAPS-containing buffer. Representative of three biological replicates. (C′) Validation of the anti-dRaptor antibody. Left panel: Kc167 cells were treated with the indicated dsRNAs for 5 days, prior to lysis. The band corresponding to the dRaptor protein, running at approximately 180 kDa, significantly decreases upon Raptor knockdown. Right panel: The Raptor band observed in a TOR immunoprecipitation runs at the same height as the Raptor band in whole-cell lysates or in Raptor immunoprecipitates. (C″) Immunoprecipitation using an unrelated anti-GFP antibody verifies that the Raptor co-immunoprecipitation is specific.

D, D′ Gene Ontology enrichment analysis using DAVID (Huang da *et al*, 2009a,b) on proteins co-immunoprecipitating with FLAG-RagA + FLAG-RagC, identified by mass spectrometry, identifies ribosomal/translation proteins as highly enriched (D). Top enriched translation-related proteins are listed in (D′) together with peptide counts from three biological replicates.

 © 2016 The Authors

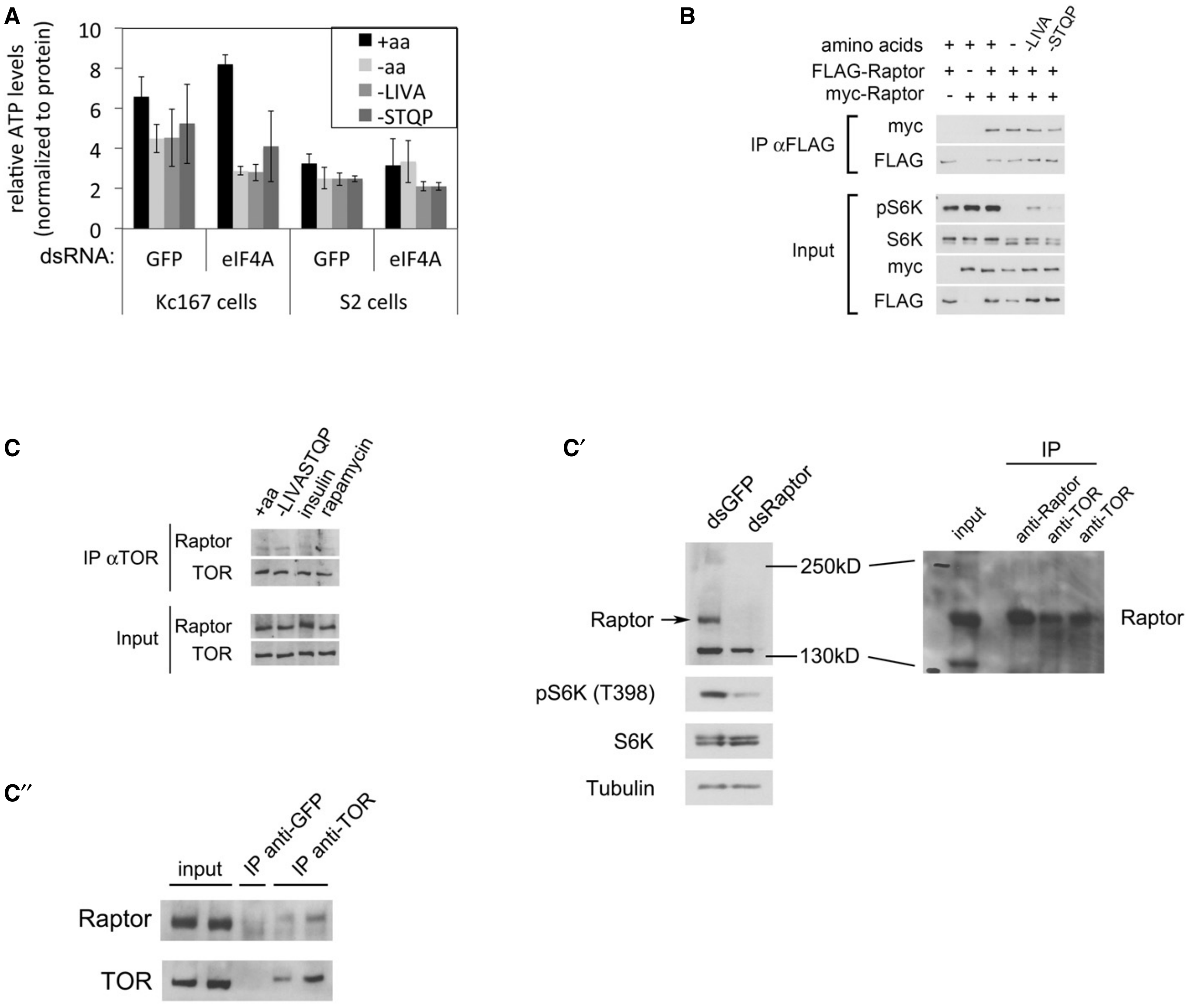

D

Annotation Cluster	Topic	p value (Benjamini corrected)
1	nucleotide binding	5×10^{-19}
2	mitotic spindle	3×10^{-16}
3	chaperone / UPR	3×10^{-9}
4	ribosome / translation	3×10^{-10}
5	ATPase	2×10^{-5}

D′

Protein	# Peptides (background subtracted) Replicate 1	Replicate 2	Replicate 3
RagC	1746	1282	1370
RagA	1661	954	537
Tor	108	76	19
Raptor	23	0	0
Ef1alpha48D	802	345	188
RpS3	277	270	182
eIF4G	162	218	46
RpL12	190	106	79
RpL23	199	51	30
RpS17	111	60	36
EF2	56	115	72
RpS15Aa	100	33	38
RpS16	104	68	46

Figure EV3.

Figure EV4. The TORC1 complex interacts physically with preinitiation complexes.

A The co-immunoprecipitation of myc-eIF4G with Flag-RagC sometimes decreases upon amino acid removal, although the effect is not very strong or consistent. Representative of two biological replicates.

B Recombinant RagC and eIF4G bind *in vitro*. An eIF4G protein fragment (aa 1,438–1,666) expressed and purified from bacteria binds GST-RagC expressed in bacteria, but not GST as a negative control. Left panel: Coomassie staining of samples from GST protein purification (pre-IPTG induction step; post-IPTG induction step, pellet and supernatant of bacterial lysate after overnight induction, GST protein fraction unbound to glutathione beads; final purified GST protein bound to glutathione beads). Right panel: Equal amounts of the indicated samples from the left panel were incubated with His-eIF4G (aa 1,438–1,666), washed, and blotted with anti-His antibody. Representative of two biological replicates.

C eIF4A and eIF3-S2, but not an unrelated protein Medea, co-immunoprecipitate with the Rag GTPases. Although in some cases, the binding between RagC and the initiation factors decreases upon amino acid removal (A), this effect is not consistently seen (C). Representative of two biological replicates.

D eIF4A binds more strongly to active-locked Rag GTPases than wild-type Rag GTPases. Kc167 *Drosophila* cells were transfected using FLAG-RagA, FLAG-RagC (WT and active-locked mutants), and HA-eIF4A expression vectors as indicated, and lysates were subjected to anti-FLAG immunoprecipitation. Note that active-locked Rag mutants coIP more eIF4A, despite being expressed at lower levels. Representative of three biological replicates.

E Schematic diagram illustrating with dashed lines the protein–protein interactions observed by co-immunoprecipitation in this study.

F RagC knockdown does not decrease the interaction between eIF4A and Raptor. Kc167 cells treated with either control or dRagC dsRNA were co-transfected to express FLAG-eIF4A together with myc-Raptor. The cells were starved for amino acids for 30 min, and binding of myc-Raptor to FLAG-eIF4A was detected by co-immunoprecipitation.

G Pulse-chase of dextran marks lysosomes and late endosomes. Cells were incubated for 1 h with 100 μg/ml dextran-pHrodo. After one rinse and 14-h incubation with normal Schneider's growth medium, 50 nM Lysotracker dye was added and cells were imaged live. Under these conditions, dextran marks mainly lysosomes (marked with Lysotracker) as well as some other structures of the endocytic pathway.

© 2016 The Authors

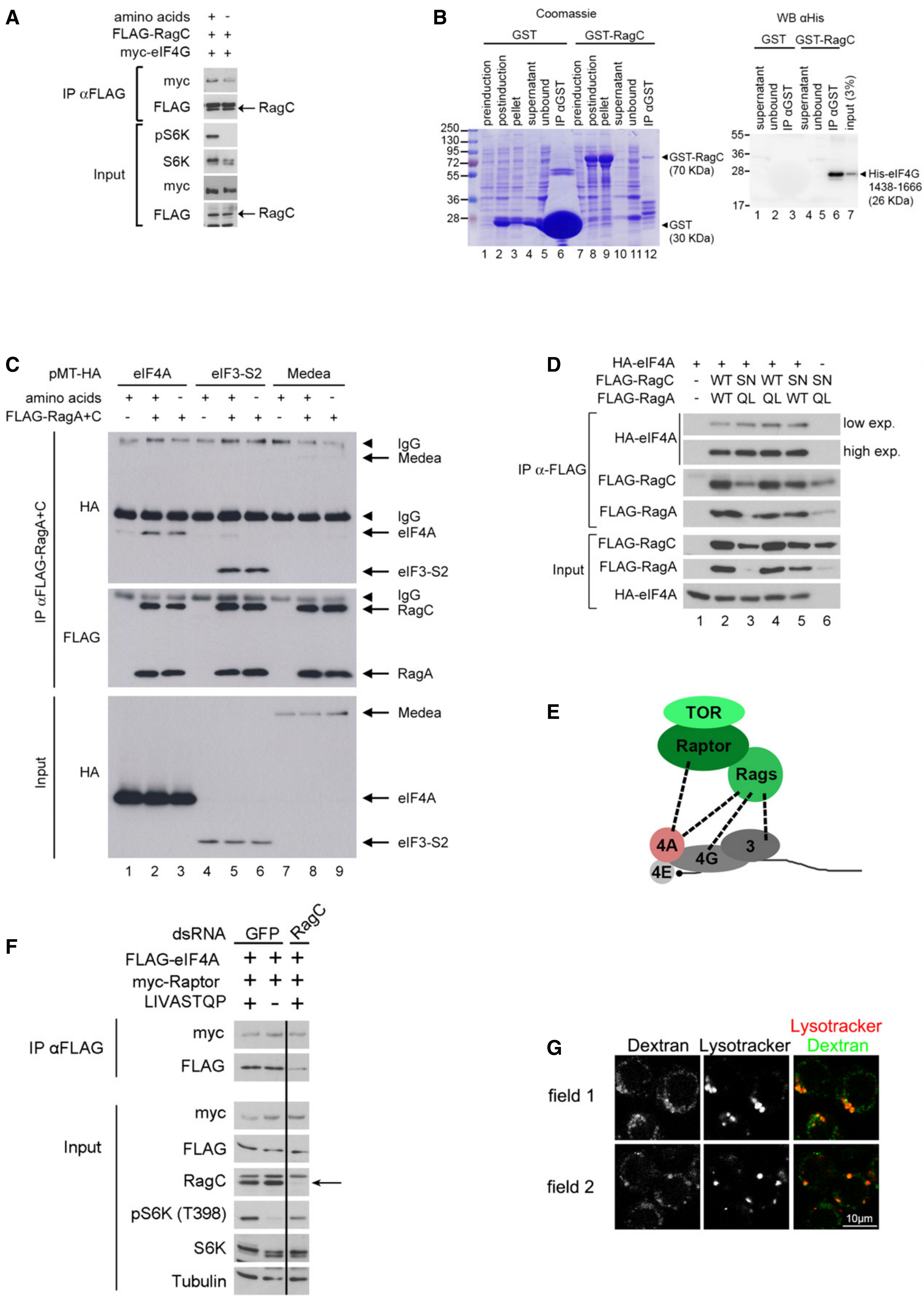
A
amino acids + -
FLAG-RagC + +
myc-eIF4G + +
IP αFLAG
myc
FLAG
RagC
Input
pS6K
S6K
myc
FLAG
RagC
B
Coomassie
GST
GST-RagC
preinduction
postinduction
pellet
supernatant
unbound
IP αGST
GST-RagC (70 KDa)
GST (30 KDa)
WB αHis
input (3%)
His-eIF4G 1438-1666 (26 KDa)
C
pMT-HA
eIF4A
eIF3-S2
Medea
amino acids
FLAG-RagA+C
IP αFLAG-RagA+C
HA
FLAG
Input
IgG
Medea
eIF4A
eIF3-S2
RagC
RagA
D
HA-eIF4A
FLAG-RagC
FLAG-RagA
WT SN WT SN SN
WT QL QL WT QL
low exp.
high exp.
IP α-FLAG
Input
E
TOR
Raptor
Rags
4A
4G
3
4E
F
dsRNA
GFP
RagC
FLAG-eIF4A
myc-Raptor
LIVASTQP
IP αFLAG
myc
FLAG
Input
RagC
pS6K (T398)
S6K
Tubulin
G
Dextran
Lysotracker
field 1
field 2
10μm

Figure EV4.

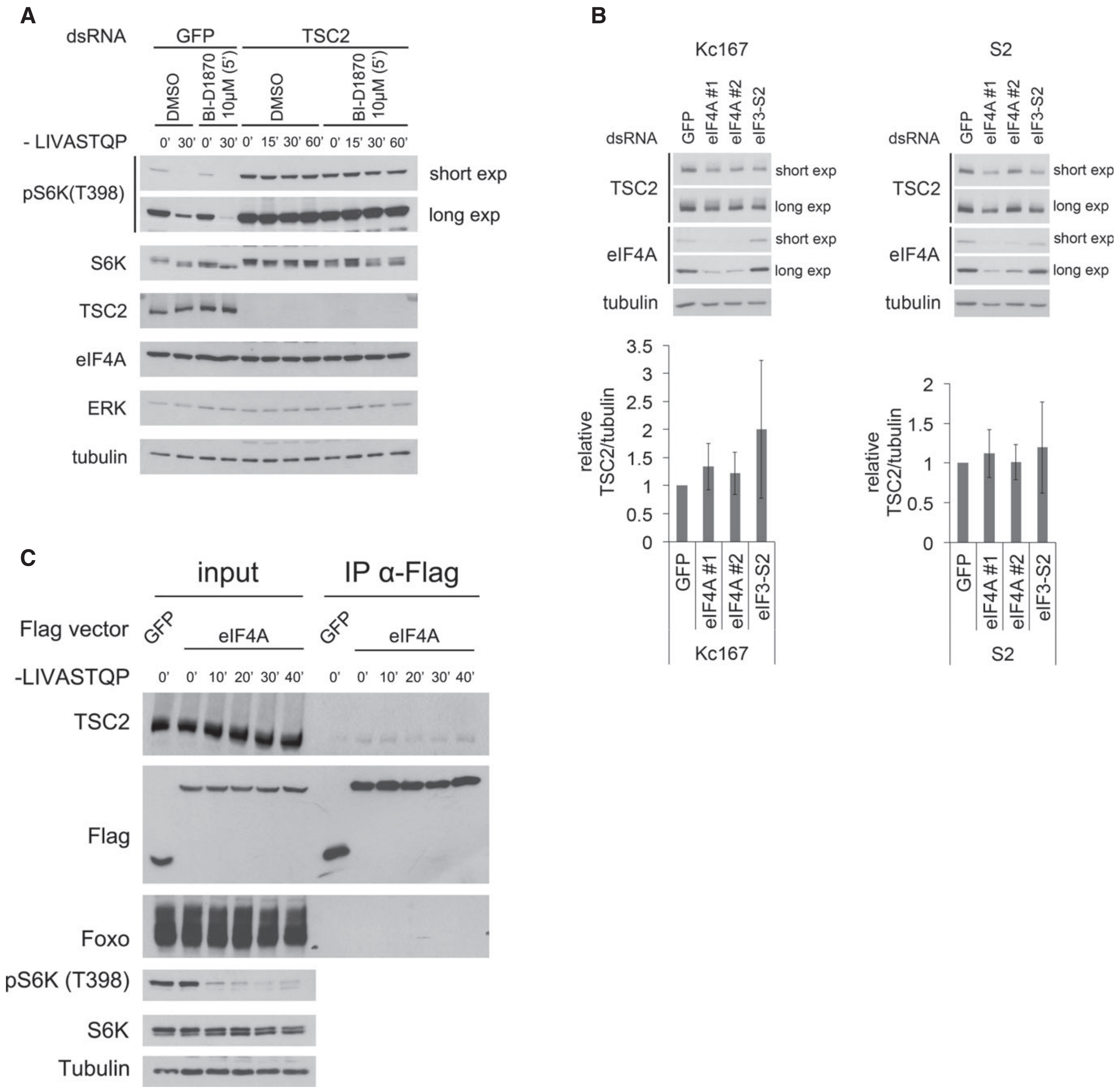

Figure EV5. Interaction between eIF4A and TSC2.

A p90RSK inhibitor BI-D1870 inhibits TORC1 in a TSC2-dependent manner. Kc167 cells were treated with dsRNA targeting TSC2 or non-targeting GFP dsRNA for 4 days, then pretreated with BI-D1870 or vehicle (DMSO) for 5 min, and then incubated in medium lacking the eight amino acids LIVASTQP for the indicated times, in the presence or absence of BI-D1870. Representative of two biological replicates.

B Knockdown of either eIF4A with two independent non-overlapping dsRNAs or of eIF3-S2 does not cause a significant drop in TSC2 protein levels normalized to tubulin levels in Kc167 or S2 cells. Error bars indicate SD. n = 3 biological replicates per cell line.

C Binding of endogenous dTSC2 to FLAG-eIF4A was detected by co-immunoprecipitation from lysates of Kc167 cell in the presence or absence of amino acids for the indicated times. As a control, an unrelated protein (Foxo) does not coIP with FLAG-eIF4A. The experiment was performed in the presence of the chemical cross-linker DSP. Representative of three biological replicates.

 © 2016 The Authors
