# Supplementary material for: eIF4A inactivates TORC1 in response to amino acid starvation
Source: EMBO J. 2016 Mar 17;35(10):1058–76. doi: 10.15252/embj.201593118 (PMC4868951; doi:10.15252/embj.201593118)
Supplement: Supplementary file 5 — Source Data for Figure 1 [file EMBJ-35-1058-s003.pdf]

Figure 1a

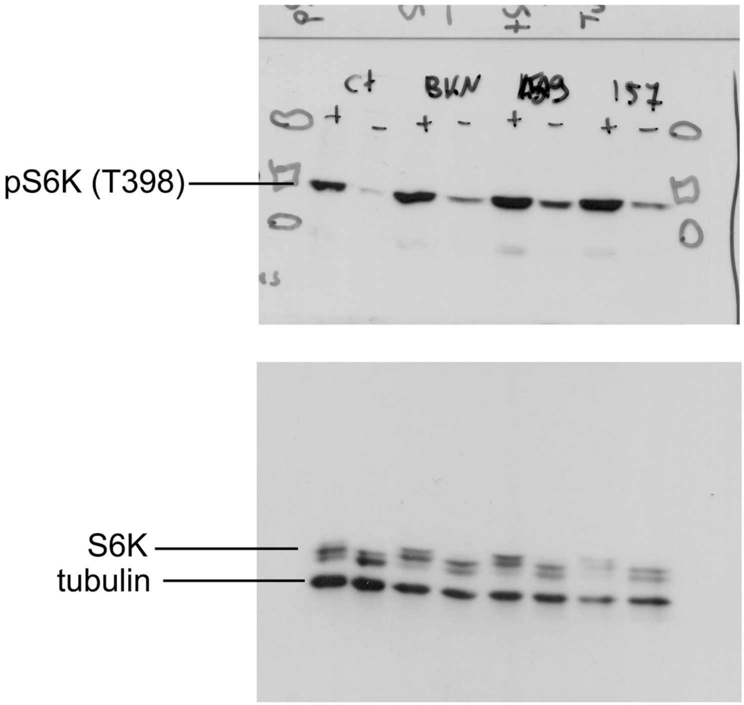

The membrane was co-incubated
with anti-S6K and anti-tubulin antibody.

Figure 1b

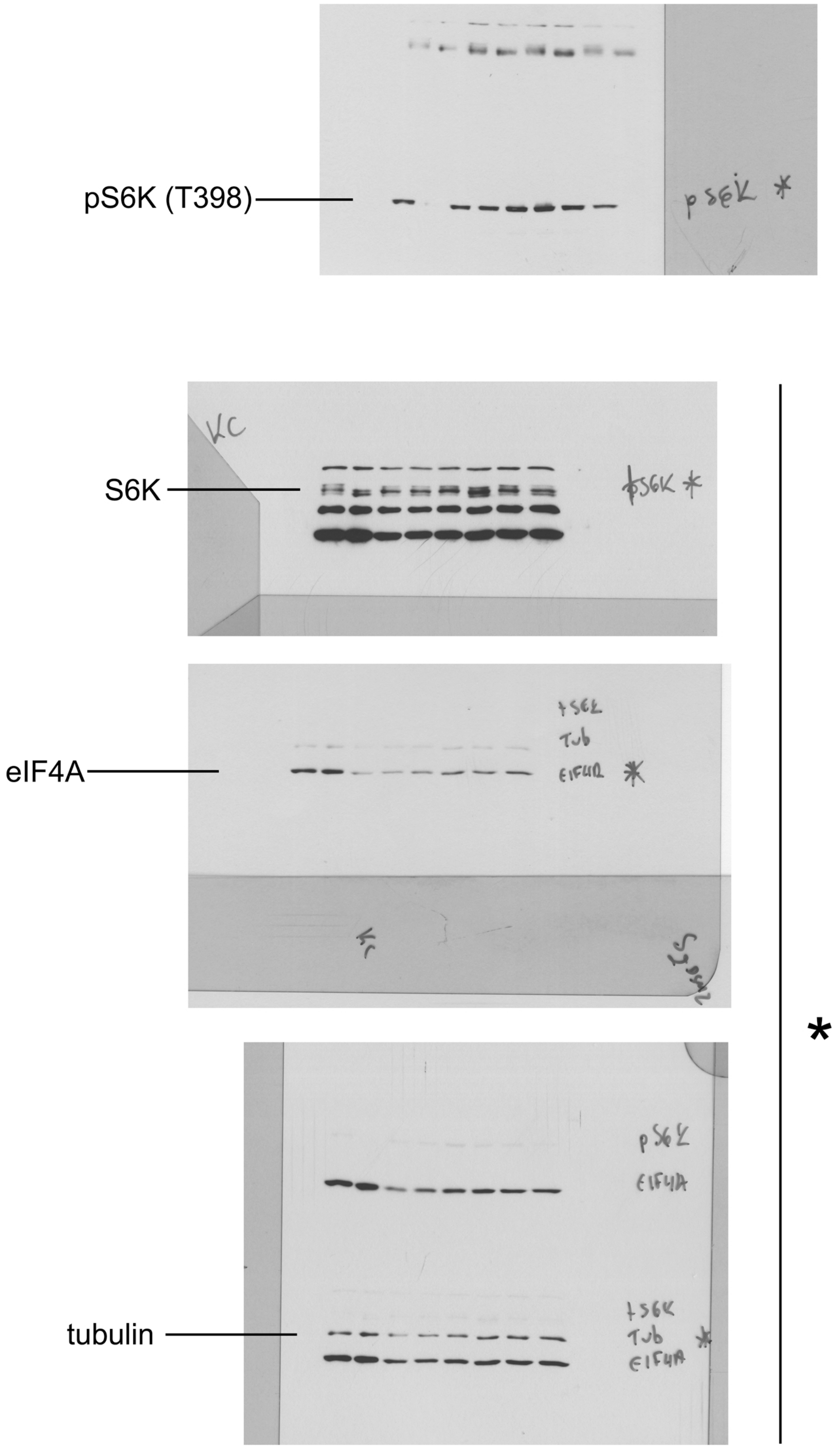

* The membrane was co-incubated with anti-S6K, anti-tubulin and anti-eIF4A and antibody. Different exposures were used for the figure.

Figure 1e

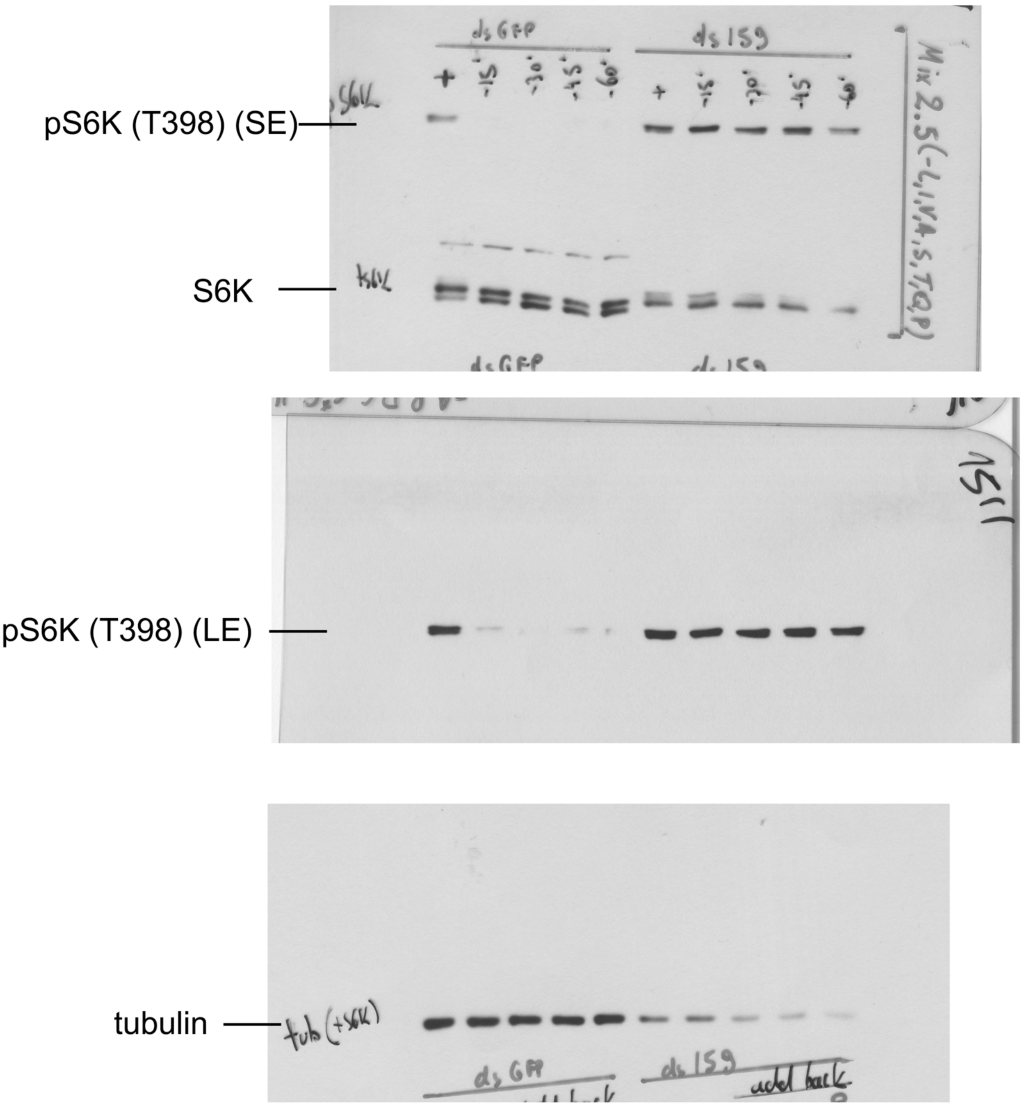

Figure 1f

*

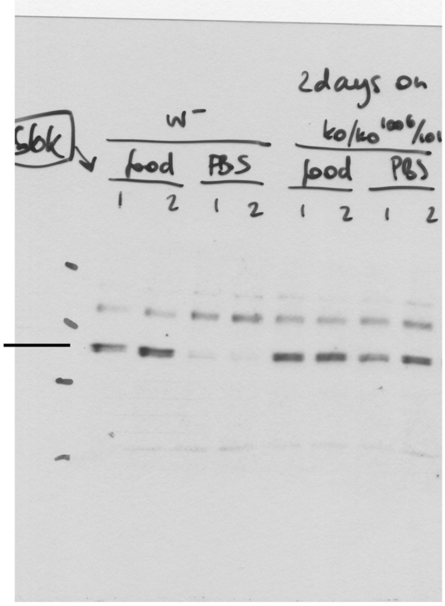

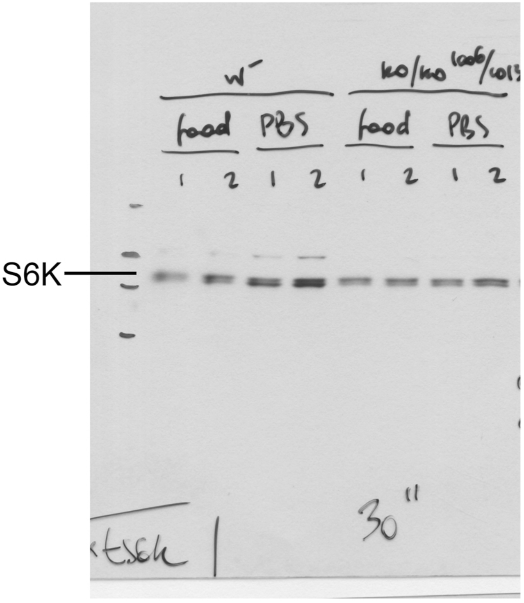

*

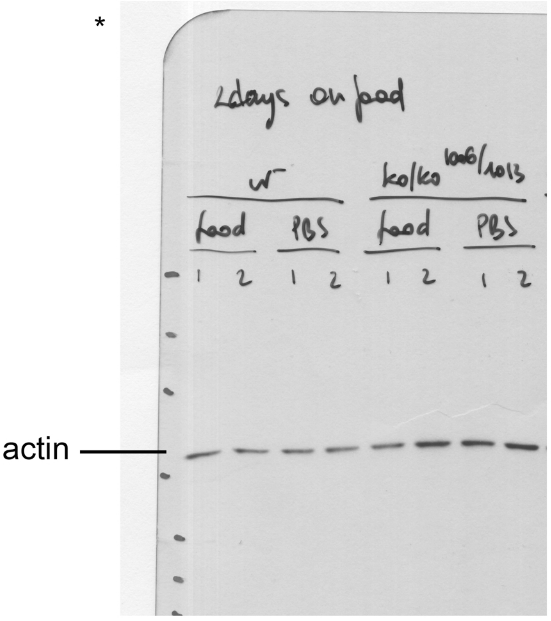

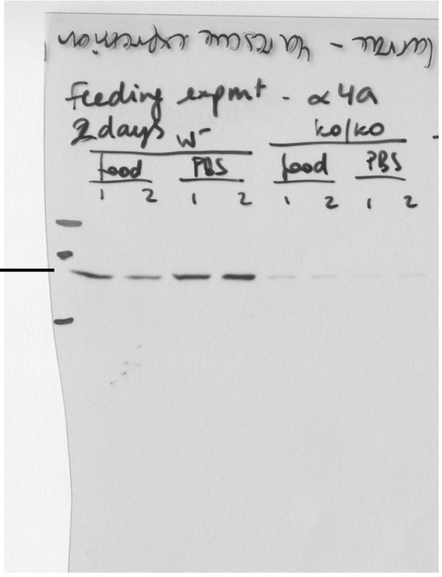

* the membrane of pS6K was reblotted with the mouse anti-actin antibody.
