# Supplementary material for: eIF4A inactivates TORC1 in response to amino acid starvation
Source: EMBO J. 2016 Mar 17;35(10):1058–76. doi: 10.15252/embj.201593118 (PMC4868951; doi:10.15252/embj.201593118)
Supplement: Supplementary file 6 — Source Data for Figure 2 [file EMBJ-35-1058-s004.pdf]

Figure 2

a

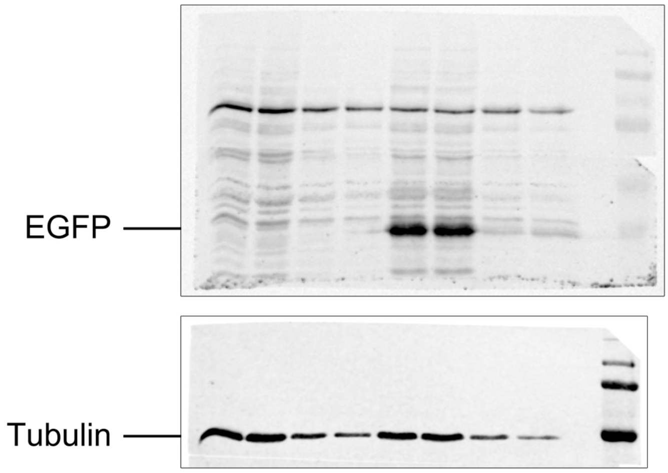

a’

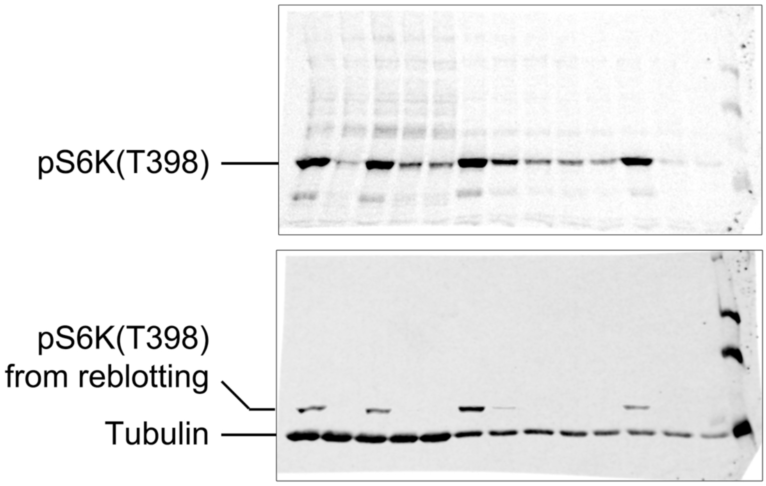

Figure 2b 1/3

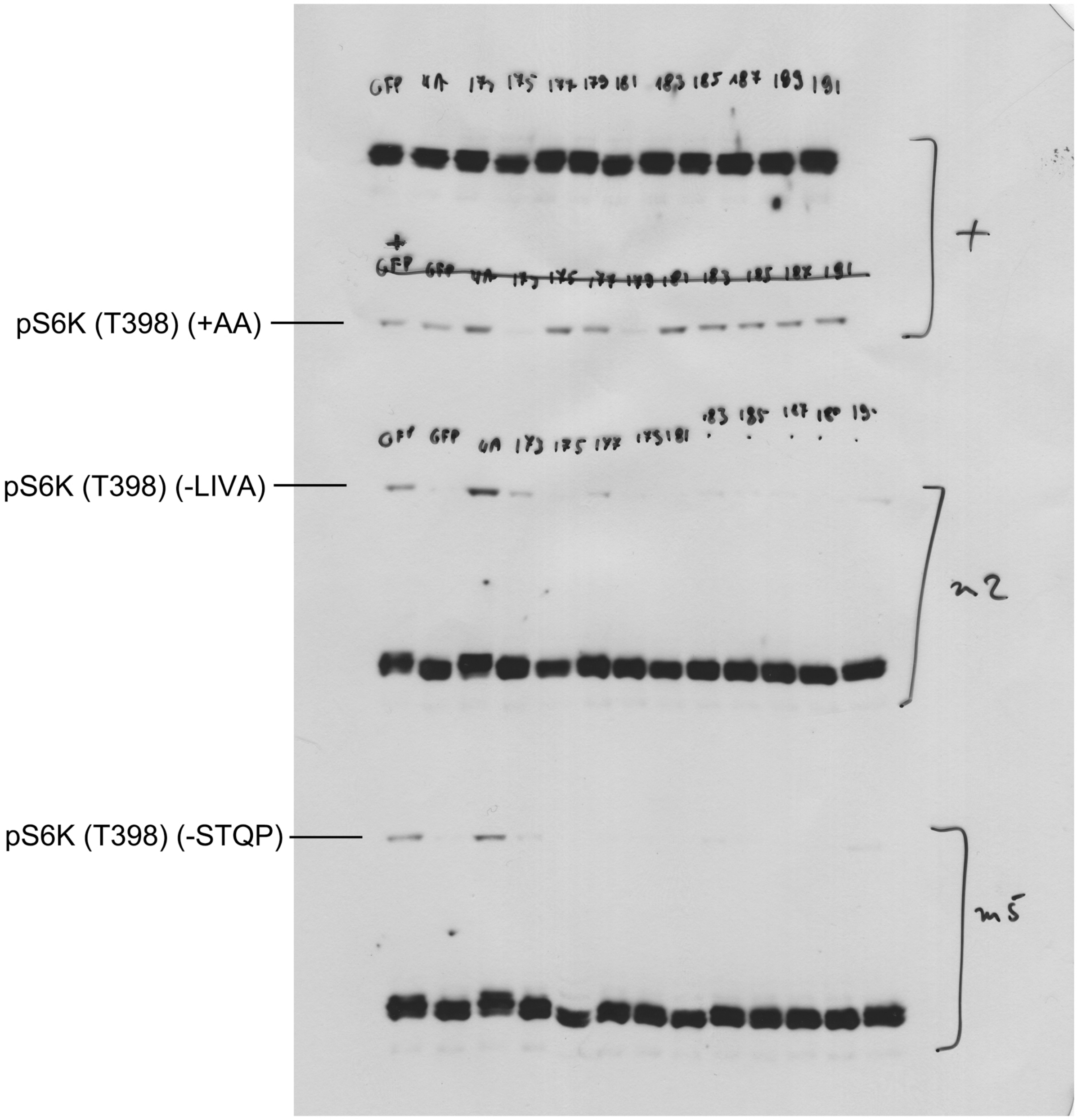

Figure 2b 2/3

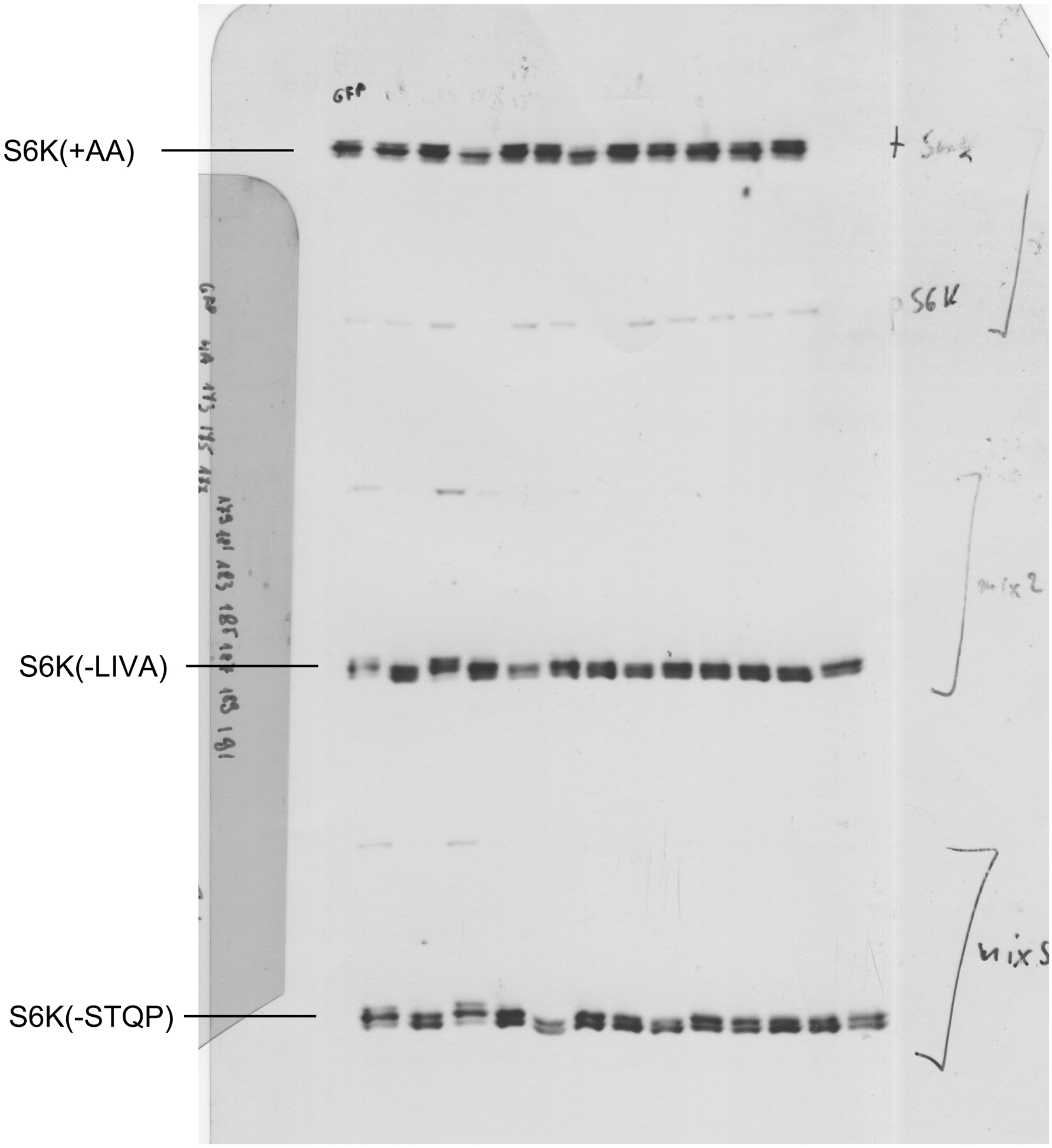

Figure 2b 3/3

Tubulin (+AA)

Tubulin (-LIVA)

Tubulin (-STQP)

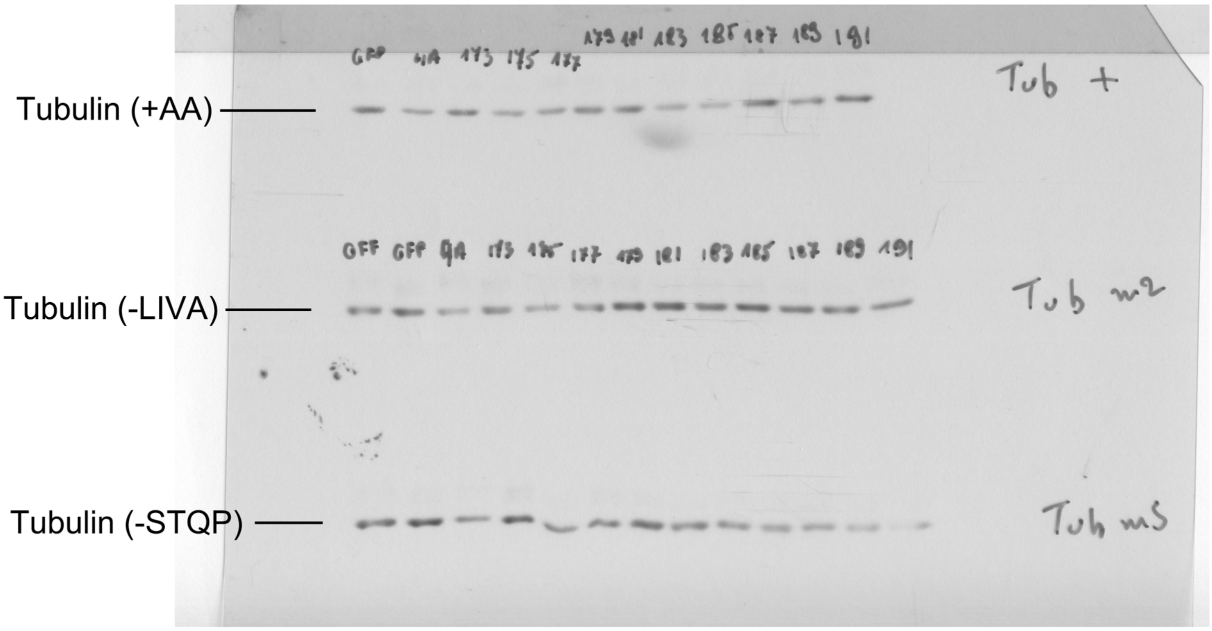

Figure 2c

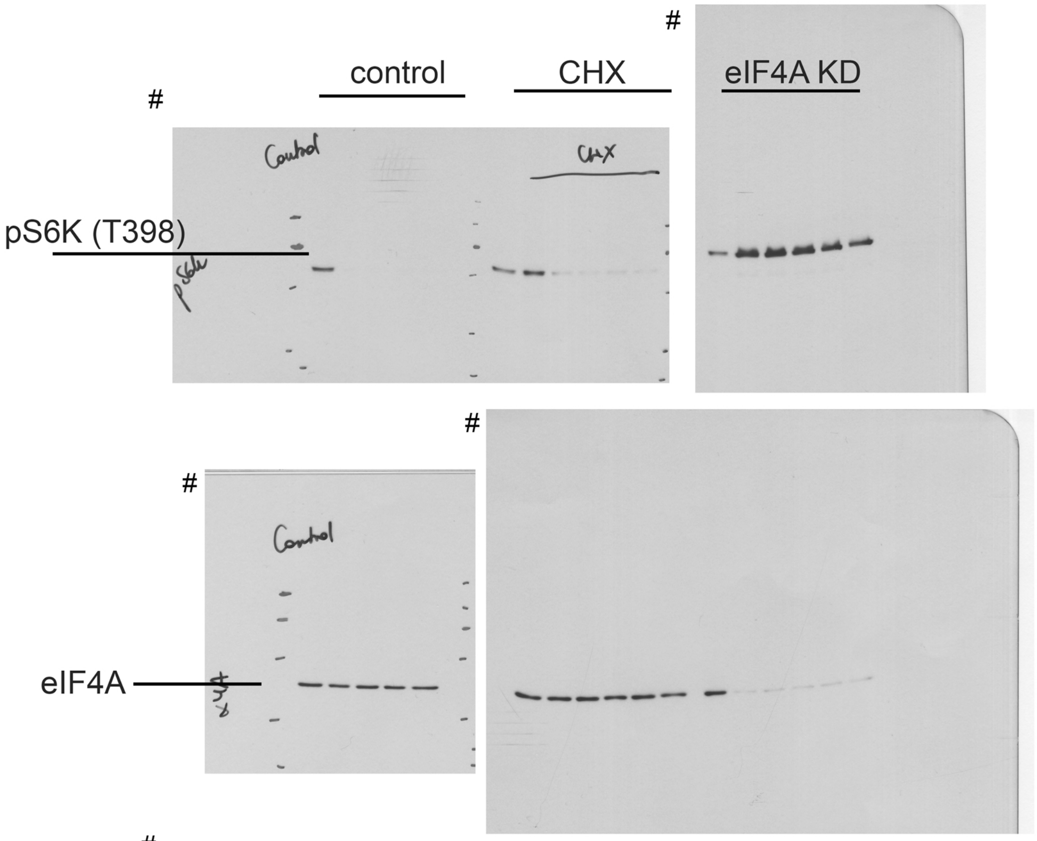

The membranes of pS6K were reblotted with the eIF4A antibody.

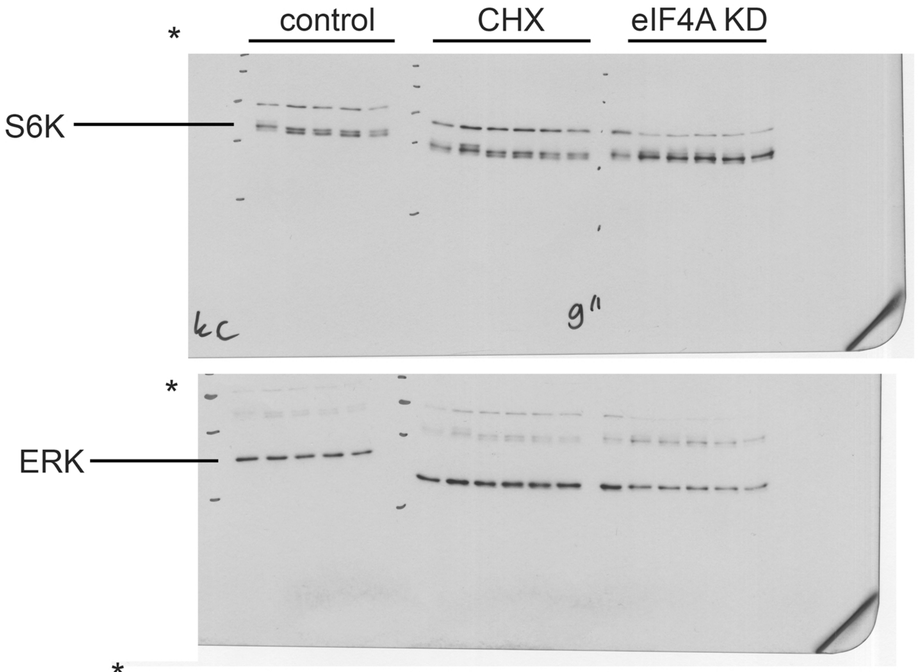

* The membranes of S6K were reblotted with the ERK antibody.
