# Supplementary figures and images for: eIF4A inactivates TORC1 in response to amino acid starvation
Source: EMBO J. 2016 Mar 17;35(10):1058–76. doi: 10.15252/embj.201593118 (PMC4868951; doi:10.15252/embj.201593118)

# Figure 3a

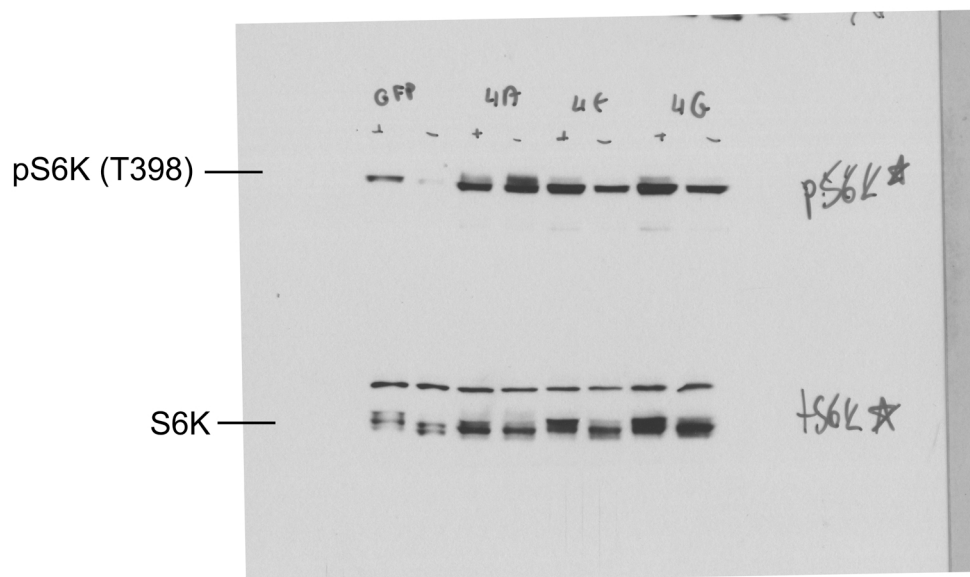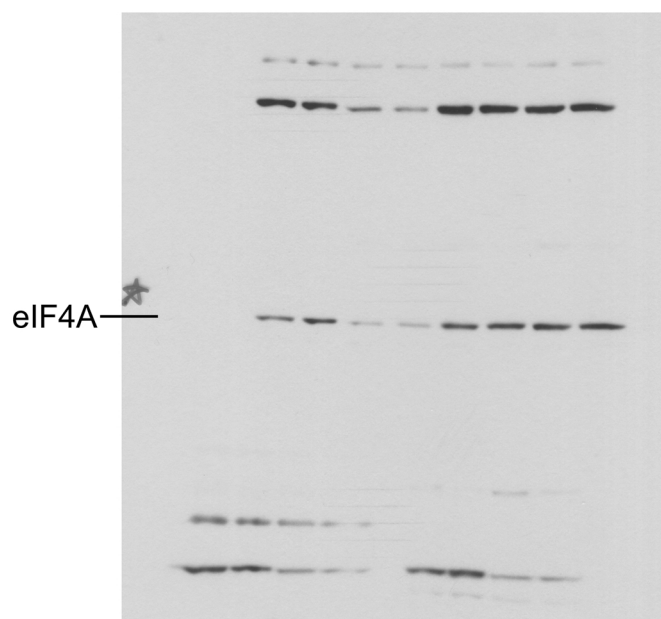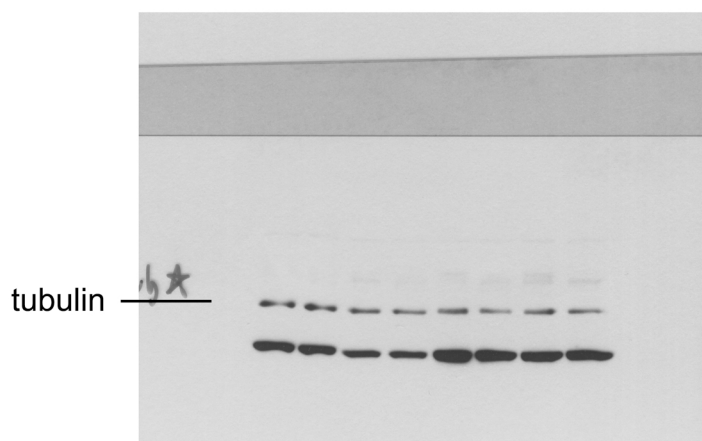

Supplement: Supplementary file 7 — Source Data for Figure 3 [file EMBJ-35-1058-s005.pdf]

# Figure 4a

Figure 4a corresponds to lanes 3-6 on all blots

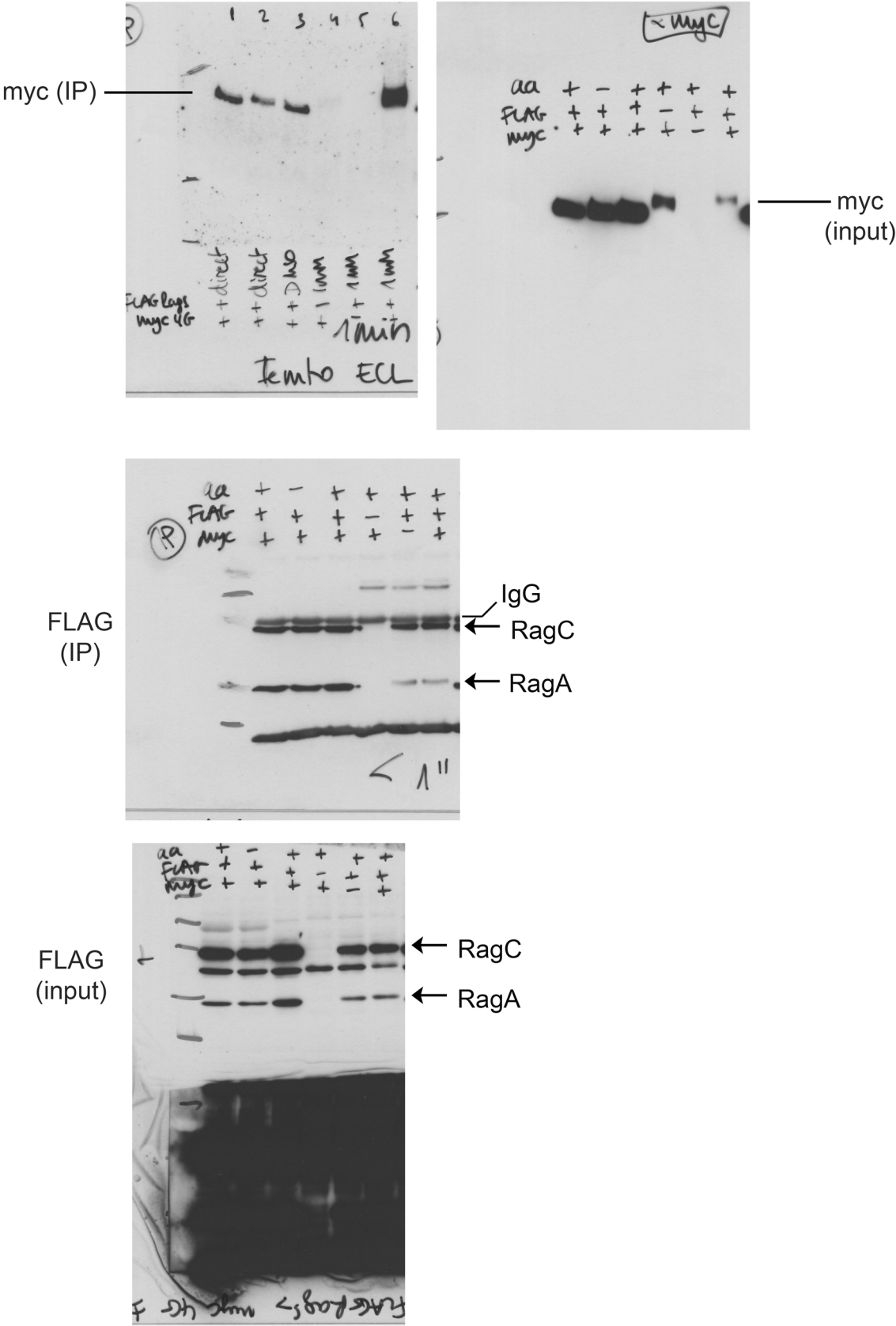



# Figure 4c

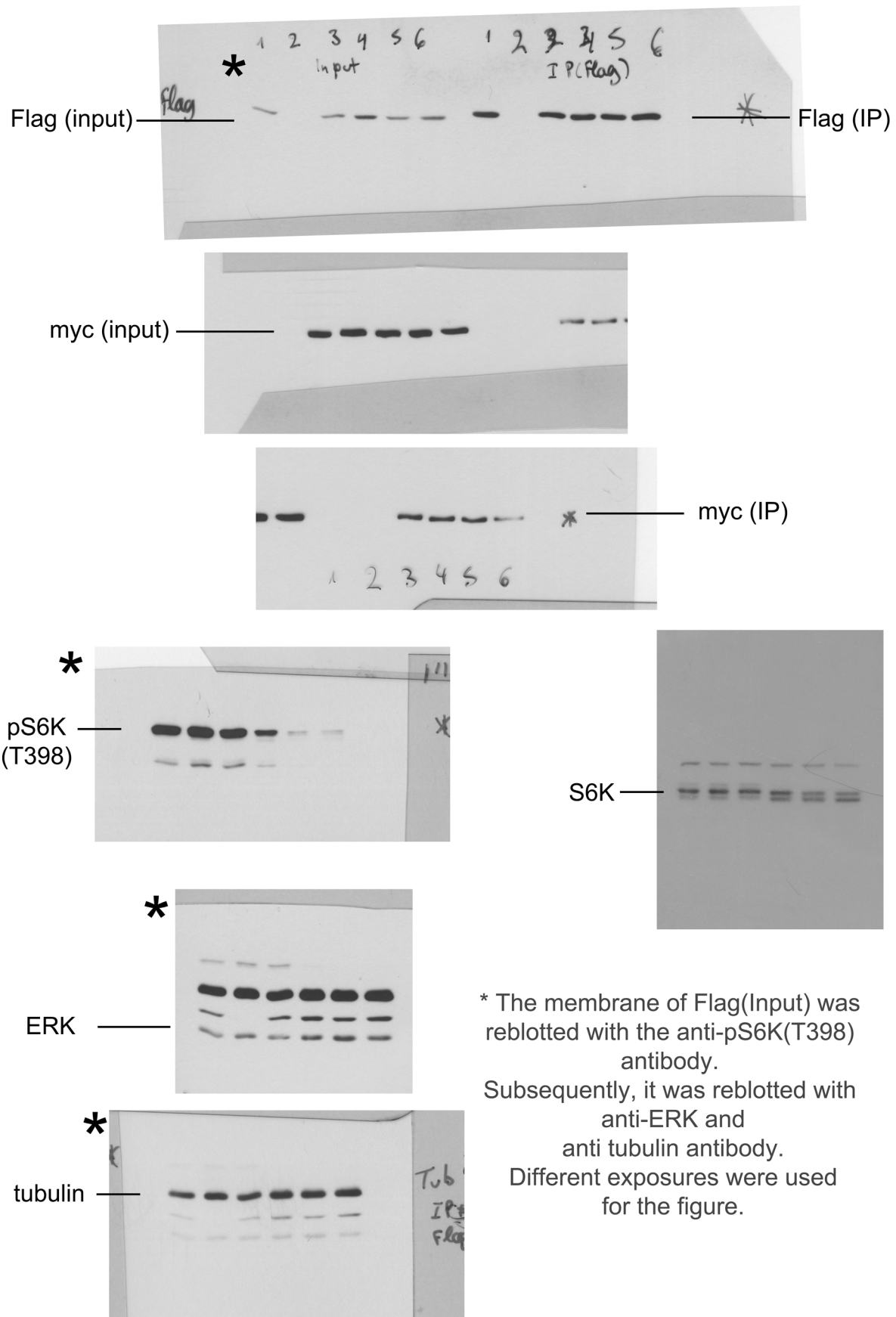

Supplement: Supplementary file 8 — Source Data for Figure 4 [file EMBJ-35-1058-s006.pdf]
