# Supplementary material for: eIF4A inactivates TORC1 in response to amino acid starvation
Source: EMBO J. 2016 Mar 17;35(10):1058–76. doi: 10.15252/embj.201593118 (PMC4868951; doi:10.15252/embj.201593118)
Supplement: Supplementary file 9 — Source Data for Figure 5 [file EMBJ-35-1058-s007.pdf]

Figure 5a

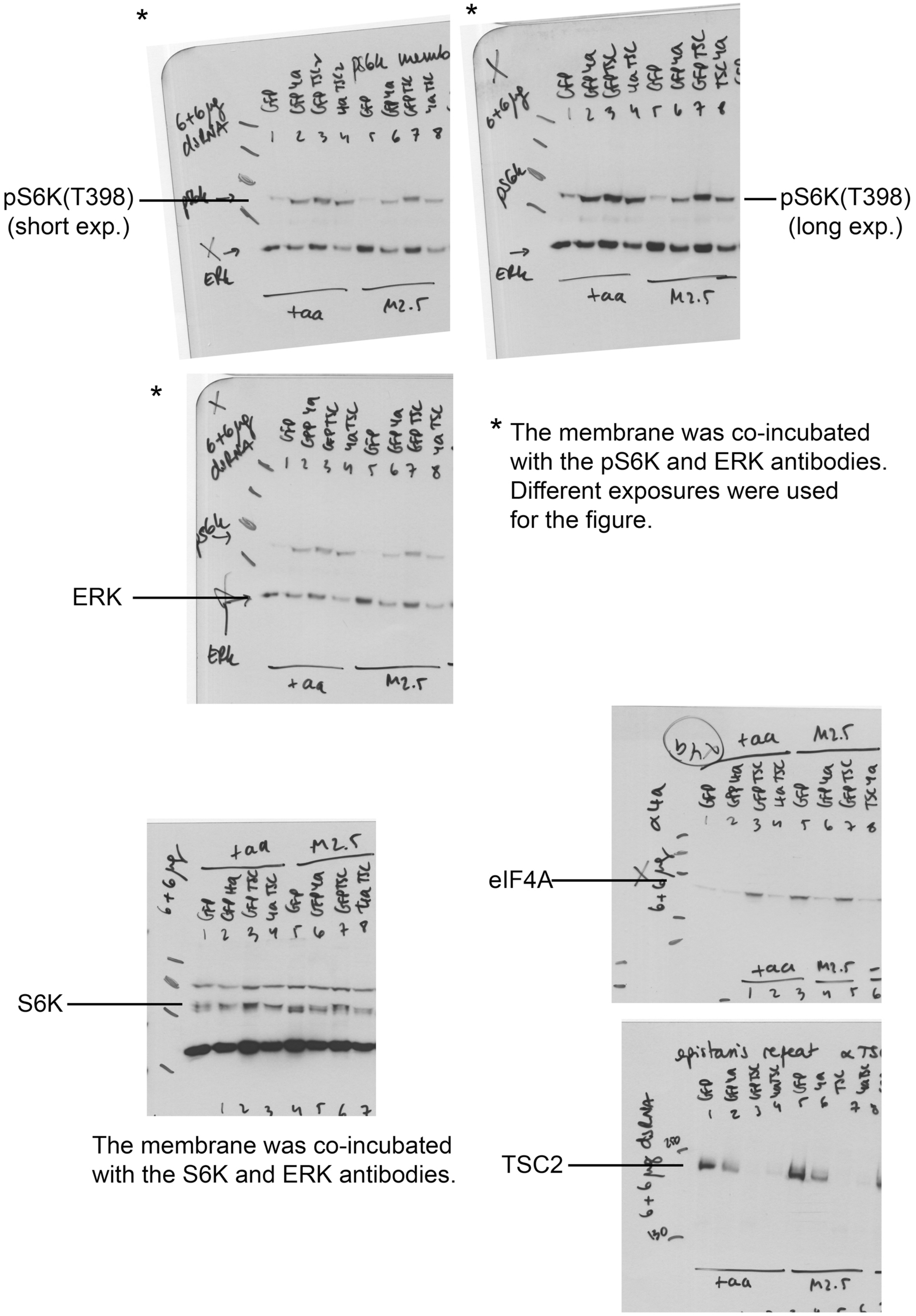

* The membrane was co-incubated with the pS6K and ERK antibodies. Different exposures were used for the figure.

The membrane was co-incubated with the S6K and ERK antibodies.

Figure 5b

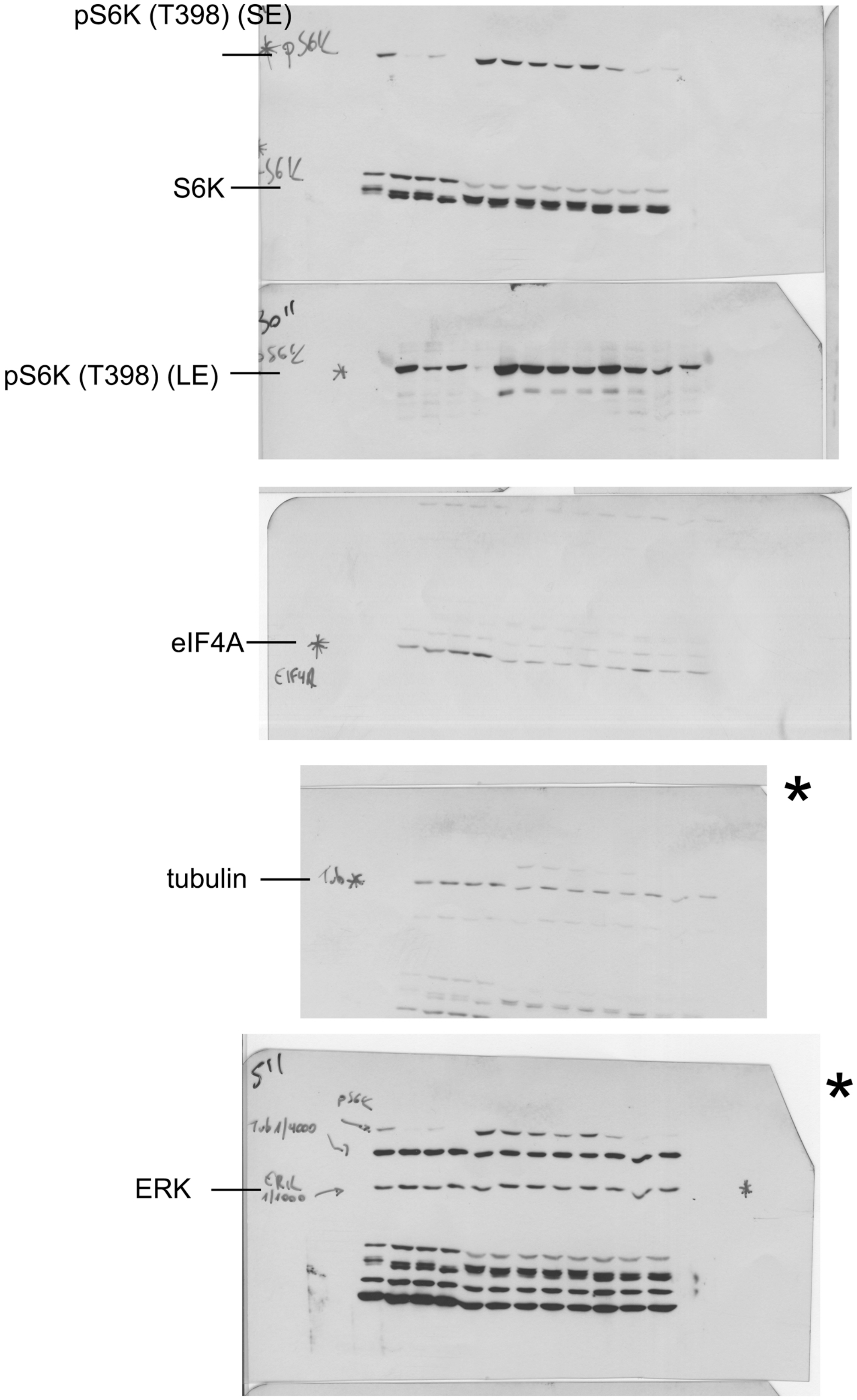

*The membrane of pS6K was
reblotted with the anti-ERK and anti-tubulin antibody

Figure 5c

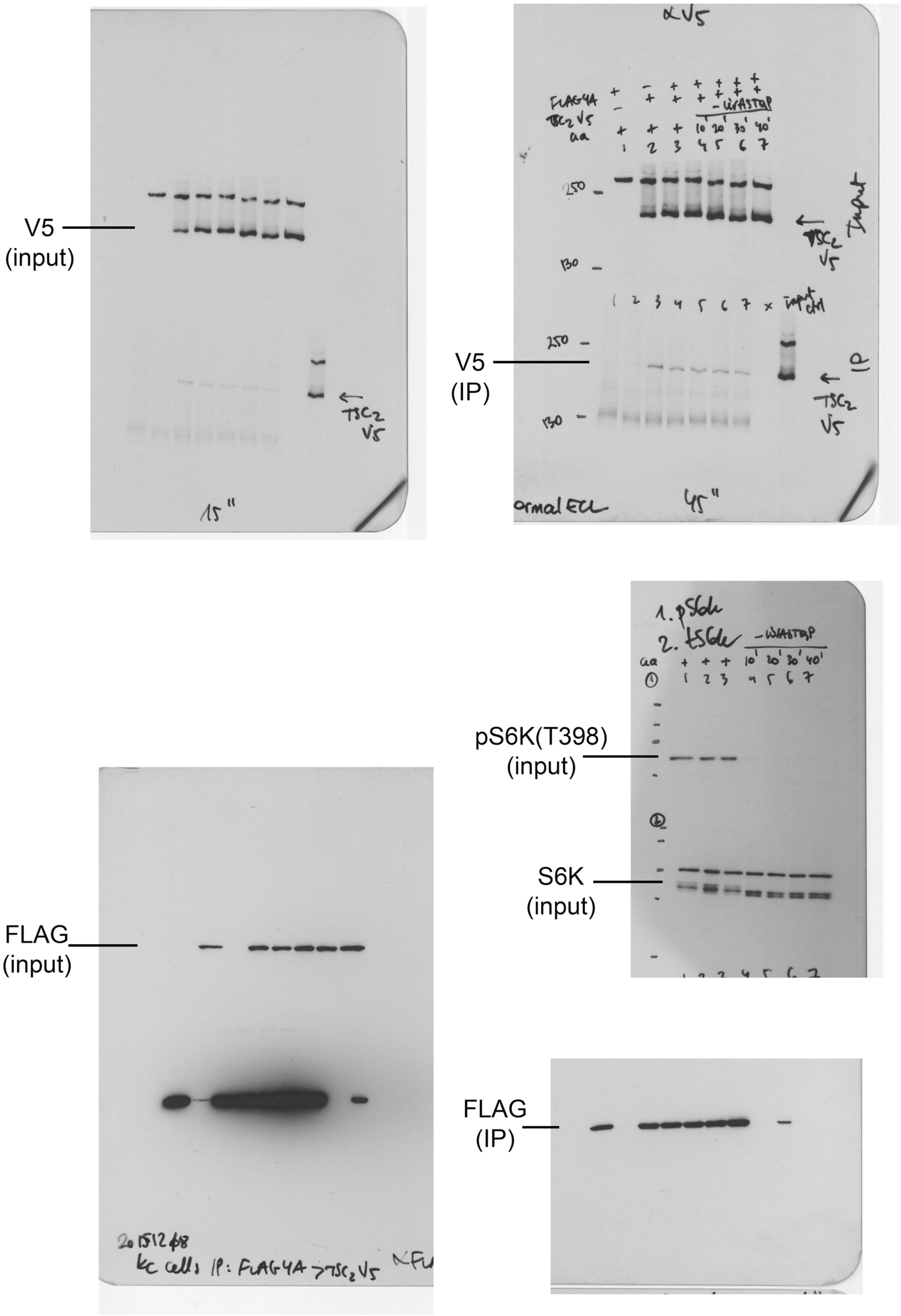

Figure 5d

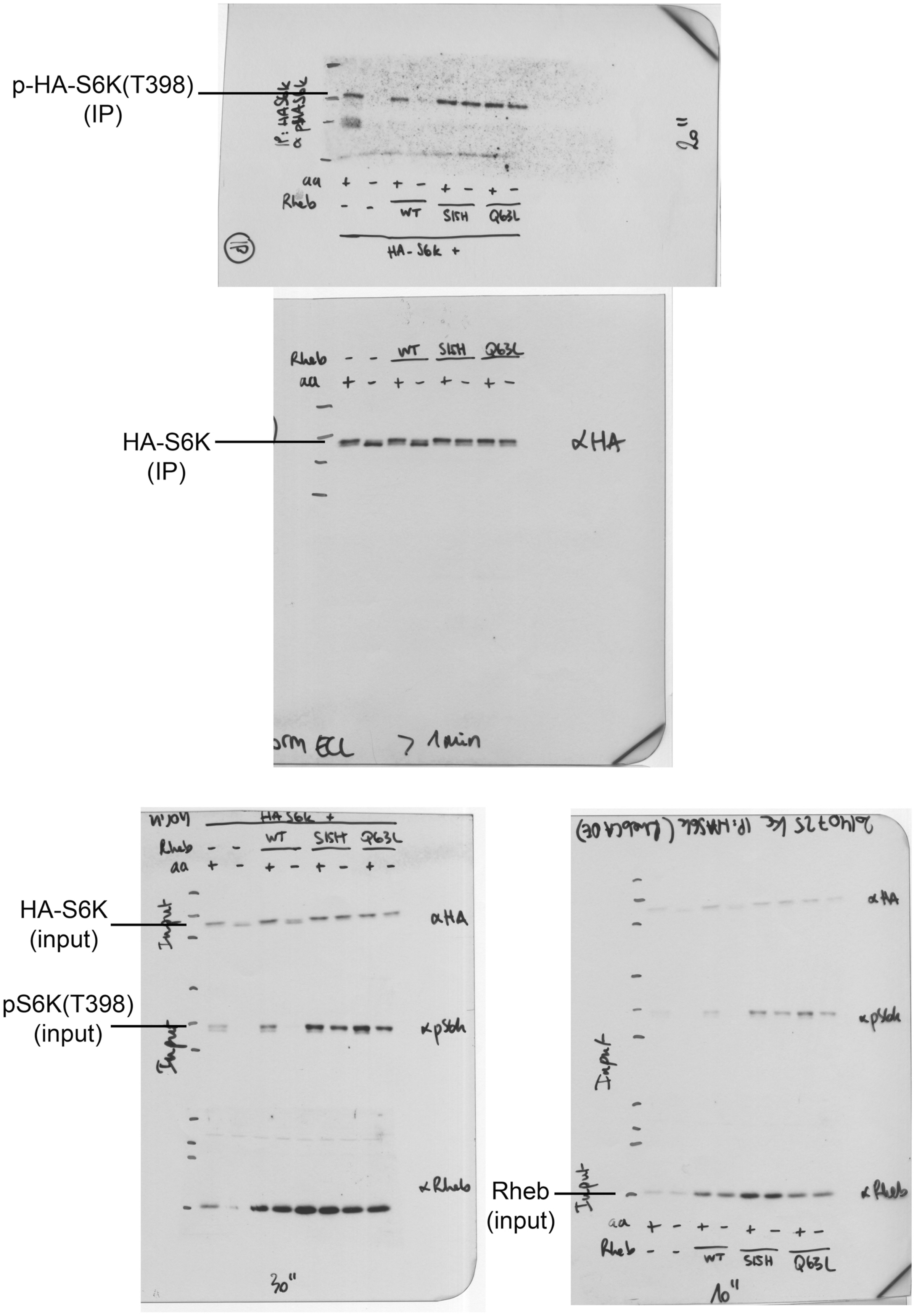
p-HA-S6K(T398)
(IP)
aa
Rheb
WT
S15H
Q63L
HA-S6k +
HA-S6K
(IP)
αHA
HA-S6K
(input)
pS6K(T398)
(input)
Rheb
(input)
αRheb
30"
10"
