# Supplementary material for: eIF4A inactivates TORC1 in response to amino acid starvation
Source: EMBO J. 2016 Mar 17;35(10):1058–76. doi: 10.15252/embj.201593118 (PMC4868951; doi:10.15252/embj.201593118)
Supplement: Supplementary file 10 — Source Data for Figure 6 [file EMBJ-35-1058-s008.pdf]

Figure 6b 1/2

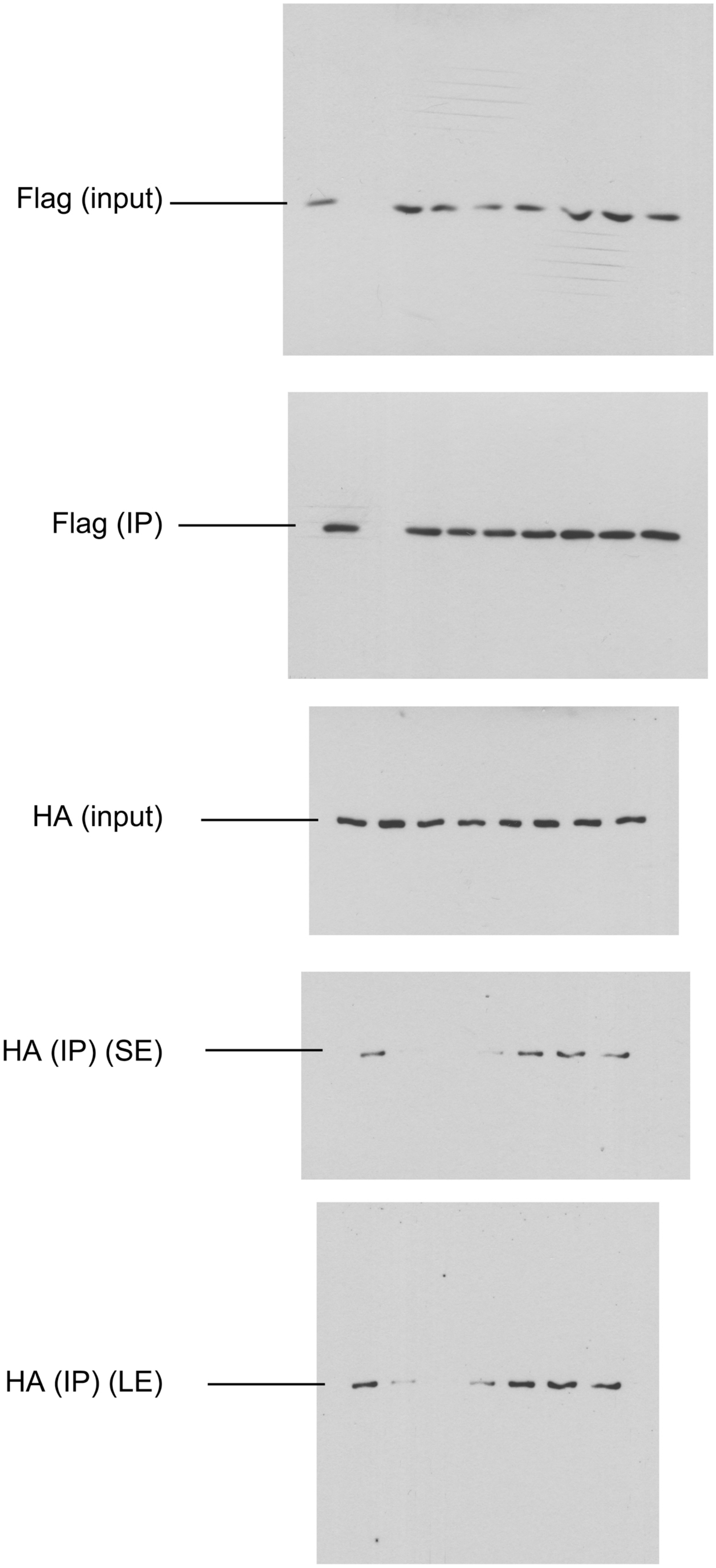

Figure 6b 2/2

pS6K (T398) (SE)

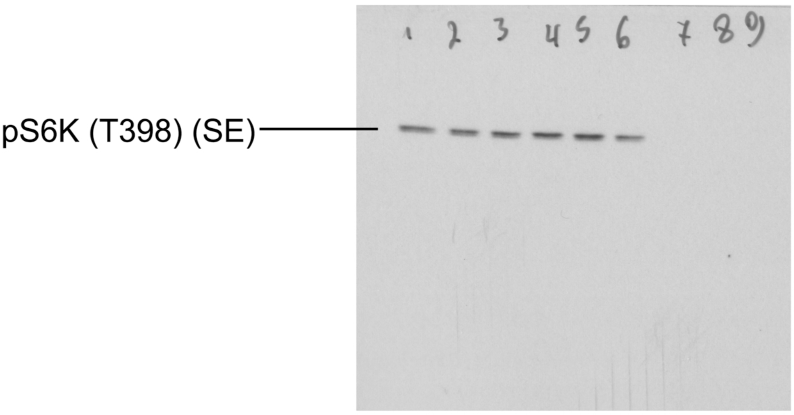

pS6K (T398) (LE)

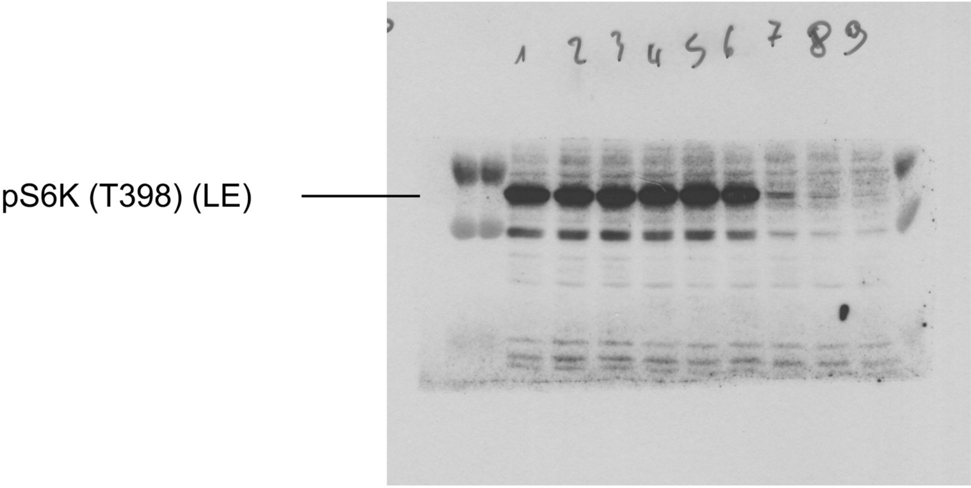

S6K

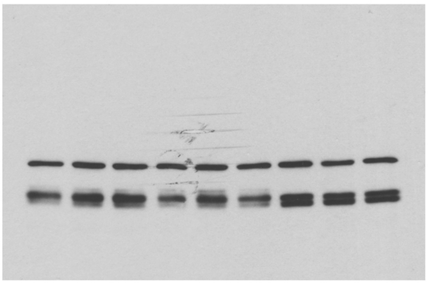

tubulin

ERK

The membrane was co-incubated with anti-tubulin and anti-ERK antibody

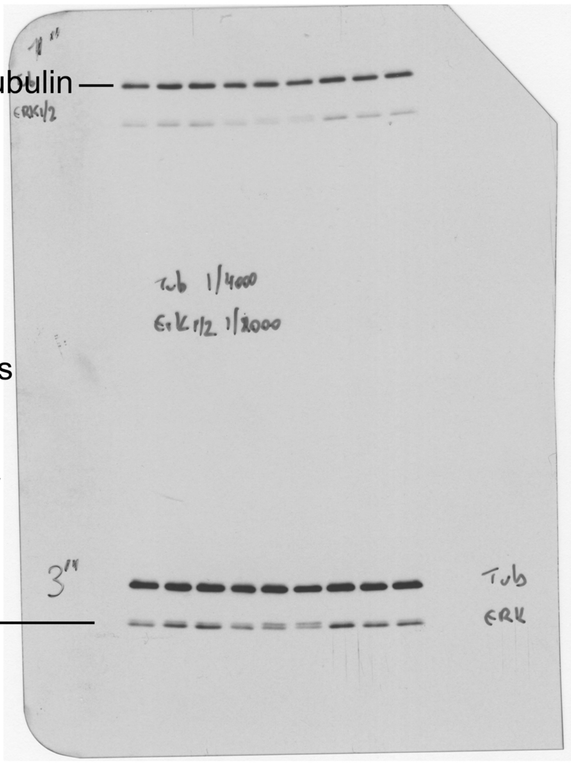

Figure 6c

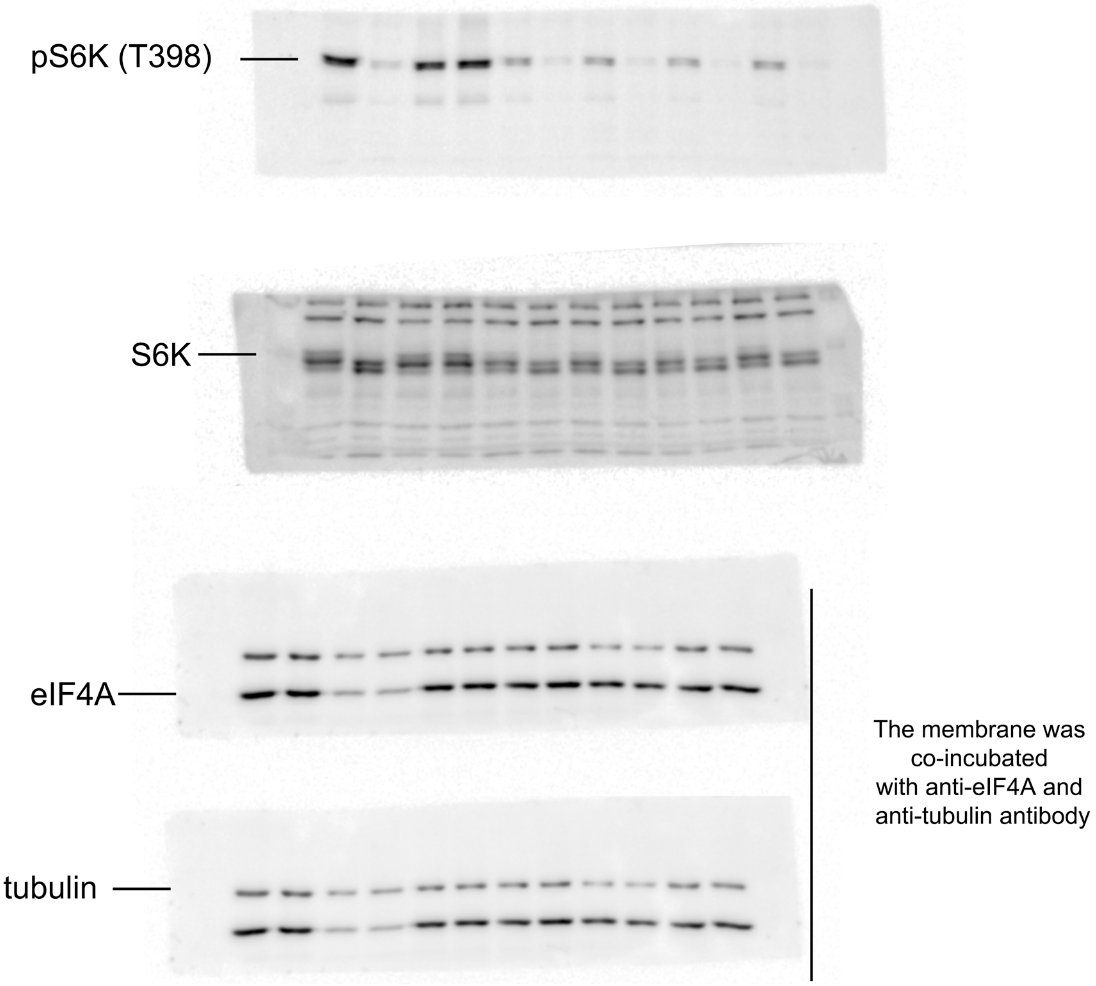
pS6K (T398)
S6K
eIF4A
tubulin
The membrane was co-incubated with anti-eIF4A and anti-tubulin antibody

Figure 6d

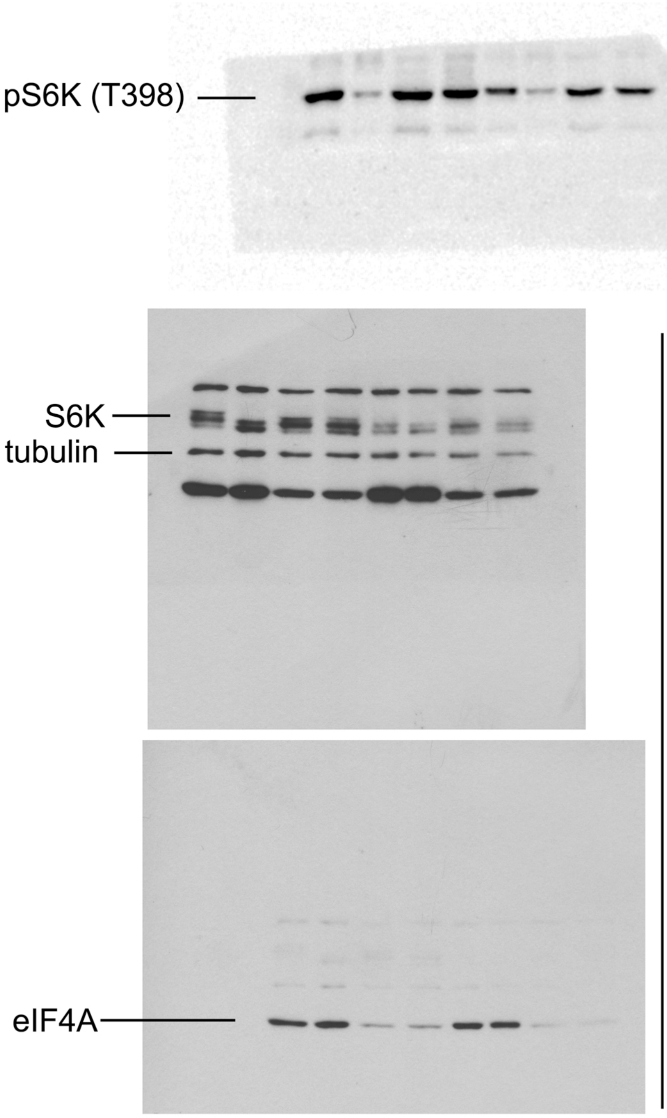

The membrane was co-incubated with anti-S6K, anti-tubulin and anti-eIF4A and antibody. Different exposures were used for the figure.

Figure 6e

pS6K T398 (LE)

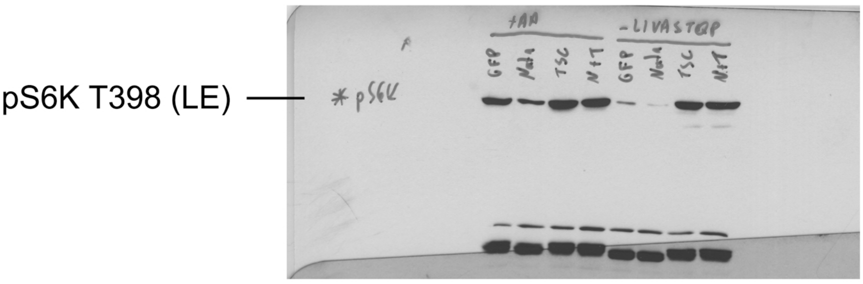

eIF4A

pS6K T398 (SE)

S6K

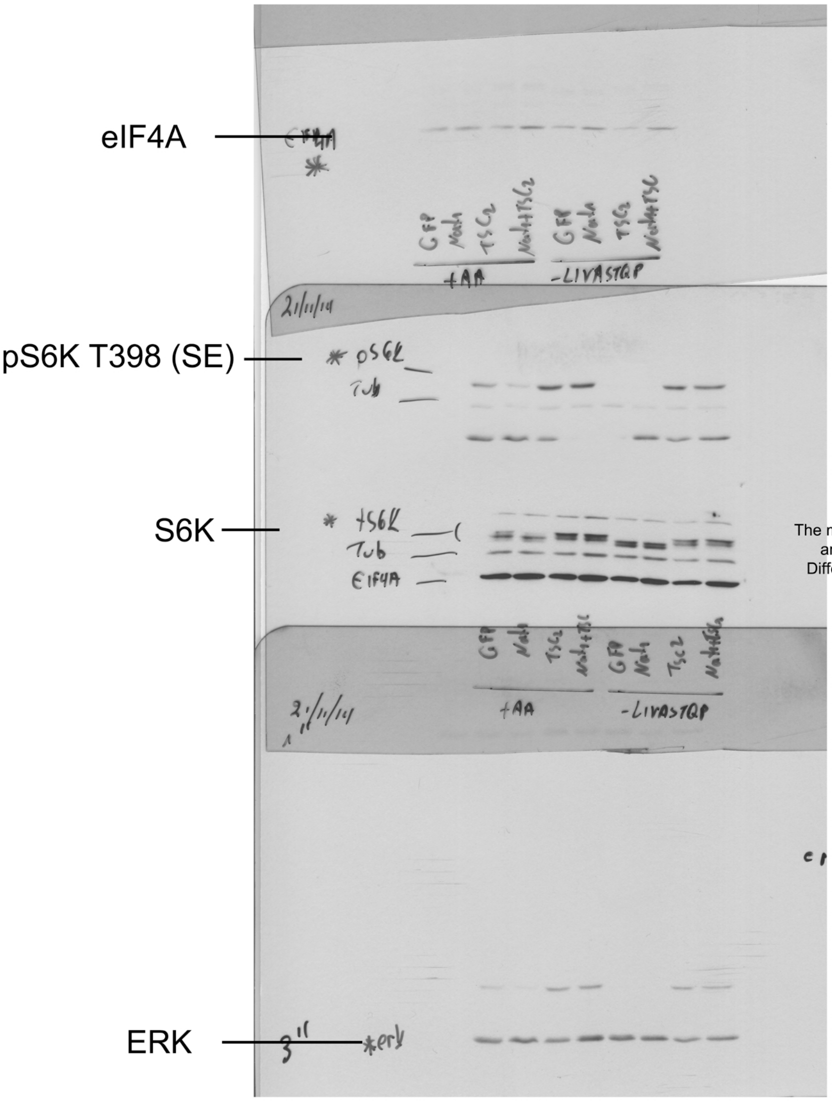

The membrane was co-incubated with anti-S6K, anti-tubulin and anti-eIF4A and antibody. Different exposures were used for the figure.

ERK

Tubulin

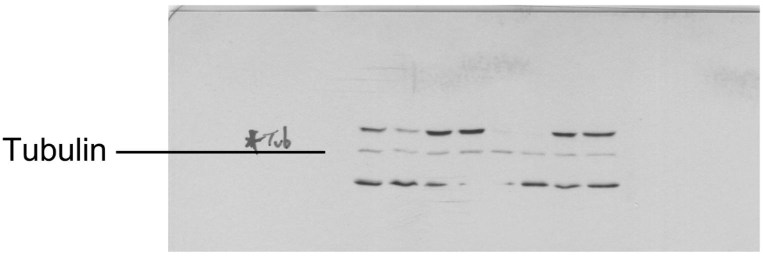

*The membrane was co-incubated with anti-pS6K (T398),anti-tubulin antibody and anti-ERK antibody. Different exposures were used for the figure.
